# 3T vs. 7T fMRI: capturing early human memory consolidation after motor task utilizing the observed higher functional specificity of 7T

**DOI:** 10.3389/fnins.2023.1215400

**Published:** 2023-08-10

**Authors:** Silke Kreitz, Angelika Mennecke, Laura Konerth, Julie Rösch, Armin M. Nagel, Frederik B. Laun, Michael Uder, Arnd Dörfler, Andreas Hess

**Affiliations:** ^1^Department of Neuroradiology, University Hospital Erlangen, Friedrich-Alexander-University Erlangen-Nürnberg (FAU), Erlangen, Germany; ^2^Institute for Pharmacology and Toxicology, Friedrich-Alexander-University Erlangen-Nürnberg (FAU), Erlangen, Germany; ^3^Institute of Radiology, University Hospital Erlangen, Friedrich-Alexander University Erlangen-Nürnberg (FAU), Erlangen, Germany; ^4^FAU NeW—Research Center for New Bioactive Compounds, Erlangen, Germany

**Keywords:** 7T fMRI, graph theory, resting state, motor task, offline replay, memory

## Abstract

**Objective:**

Functional magnetic resonance imaging (fMRI) visualizes brain structures at increasingly higher resolution and better signal-to-noise ratio (SNR) as field strength increases. Yet, mapping the blood oxygen level dependent (BOLD) response to distinct neuronal processes continues to be challenging. Here, we investigated the characteristics of 7 T-fMRI compared to 3 T-fMRI in the human brain beyond the effect of increased SNR and verified the benefits of 7 T-fMRI in the detection of tiny, highly specific modulations of functional connectivity in the resting state following a motor task.

**Methods:**

18 healthy volunteers underwent two resting state and a stimulus driven measurement using a finger tapping motor task at 3 and 7 T, respectively. The SNR for each field strength was adjusted by targeted voxel size variation to minimize the effect of SNR on the field strength specific outcome. Spatial and temporal characteristics of resting state ICA, network graphs, and motor task related activated areas were compared. Finally, a graph theoretical approach was used to detect resting state modulation subsequent to a simple motor task.

**Results:**

Spatial extensions of resting state ICA and motor task related activated areas were consistent between field strengths, but temporal characteristics varied, indicating that 7 T achieved a higher functional specificity of the BOLD response than 3 T-fMRI. Following the motor task, only 7 T-fMRI enabled the detection of highly specific connectivity modulations representing an “offline replay” of previous motor activation. Modulated connections of the motor cortex were directly linked to brain regions associated with memory consolidation.

**Conclusion:**

These findings reveal how memory processing is initiated even after simple motor tasks, and that it begins earlier than previously shown. Thus, the superior capability of 7 T-fMRI to detect subtle functional dynamics promises to improve diagnostics and therapeutic assessment of neurological diseases.

## Highlights

- 7 T fMRI show higher functional specificity to detect the underlying neuronal signal.
- During rest after a motor task, only 7 T fMRI show enhanced motor cortex connectivity.
- The functional connections of this “offline replay” are linked to memory circuits.

## 1. Introduction

Magnetic-resonance-imaging (MRI), the gold standard in medical research and clinical diagnostics, generates high resolution images that help diagnose brain injuries, such as aneurysms, stroke, and traumatic brain injury, but also tumors and nervous system disease. With the recent clinical approval of ultra-high field 7 Tesla (7 T) magnets in 2017, hopes are arising that this technological advance would significantly improve static MRI diagnostics but also functional MRI (fMRI). Specifically, because fMRI detects a blood-oxygen-level-dependent (BOLD) signal coupled to underlying neuronal activity, it could in principle be applied toward mapping and diagnosing human brain function in a range of nervous system indications. In clinical context, monitoring resting-state inter-regional neural activity correlations (rs-fMRI) has several advantages over task-fMRI (mapping static neural activity following a specific motor or sensory task): rs-fMRI data acquisition is less complex, less time consuming and does not need patient’s cooperation. However, the implication of rs-fMRI in clinical practice is currently limited to pre-surgical planning, largely due to the increased complexity required for mapping functional connectivity and analyzing single subjects (O’Connor and Zeffiro, 2019).

In principle, a stronger magnetic field that improves upon signal-to-noise ratio (SNR) and contrast-to-noise ratio (CNR), enables imaging at higher spatial resolution with sufficient SNR and, thus the visualization of finer structures. Accordingly, BOLD 7 T-fMRI allows detection of increased BOLD contrast due to a shortened T2* relaxation (the decay of transverse magnetization; an important image contrast determinant; Yacoub et al., 2001; Peters et al., 2007), with higher spatial specificity (the intra-vascular signal contribution from draining veins is reduced; Gati et al., 1997; Duong et al., 2003).

On the other hand, higher field strengths result in increased physiological noise that dominates the temporal SNR (tSNR), especially at larger voxel sizes. However, at high image SNR, which is determined by larger voxel sizes, the tSNR reaches a plateau, limiting the benefits of increased tSNR to high spatial resolutions (Triantafyllou et al., 2005). In this study, the voxel sizes were chosen such that images at both field strengths were dominated by thermal rather than physiological noise and exhibited a tSNR below the plateau.

However, 7 T-fMRI also has disadvantages such as increased inhomogeneity in the static (B0) magnetic field, which can cause susceptibility-induced distortions that lead to degradation of image quality in a variety of applications. In addition, the shortened T2* relaxation can result in increased blurring in EPI readouts.

Since the motor cortex is less influenced by imaging-associated technical artefacts such as reduced transmit field strength, given its superficial location and proximity to the detection coil, motor tasks are frequently used to investigate the field strength influence on neuronal activation in healthy subjects (Schafer et al., 2008; van der Zwaag et al., 2009) and tumor patients (Beisteiner et al., 2011). Indeed, at higher field strength, increased BOLD signal, higher amplitudes, and average *t*-scores as well as increased activated volume have been reported. However, whether a 7 T-fMRI analysis of the resting-state connectivity in and of itself, would have potential diagnostic capability, remains largely unexplored. Earlier studies reported that 7 T, but not 3 T, by significantly higher temporal SNR ratio, improves upon spatial specificity in connected areas. This improvement enabled detection of functional connectivity (FC) that differed in the ventral tegmental area of patients with depression relative to healthy controls (Hale et al., 2010; Morris et al., 2019). However, assessing whether a motor task directly impacts on subsequent resting-state connectivity is beyond the detection limit of 3 T-fMRI, and have not yet been demonstrated for 7 T-fMRI.

Through the use of invasive electrophysiological methods, the existence of ongoing neuronal firing immediately after a motor task has been demonstrated in animals (Gulati et al., 2014; Ramanathan et al., 2015; Yu et al., 2021). This so-called neuronal “offline replay” is presumed to be directly linked to memory encoding, as suppression of the offline replay results in poorer memory performance (Yu et al., 2021). Recently, learning-related offline replay in the human brain was reported in a pilot trial in which participants were implanted with intracortical microelectrode arrays (Eichenlaub et al., 2020), providing proof of principle that early memory encoding in the resting-state can be detected.

Focusing on the human visual cortex, that is known to be easily and strongly excitable, this principle concept of detecting neuronal replay by (3 T) fMRI was very recently shown using task-related stimuli with various inter-stimulus intervals. Frequency spectra analysis of probabilistic patterns in pre- and post-task resting-states revealed differences at frequencies indicative for the fastest and slowest stimulus presentation speed, pointing indirectly toward a replay of activation patterns in the visual cortex (Wittkuhn and Schuck, 2021). In addition to persisting activation patterns, the “replay” of task-related neuronal activity is thought to contribute also to the reconfiguration of memory-related functional connectivity across the brain (Albert et al., 2009a; Tambini et al., 2010). For 3 T-fMRI, functional replay was demonstrated during slow-wave-sleep in humans via cue-induced replay of event-related brain activation after declarative memory-encoding tasks (Rasch et al., 2007; Berkers et al., 2018). Occurrence and strength, especially of hippocampal replay patterns, were correlated with subsequent memory performance (Tambini and Davachi, 2013; Schapiro et al., 2018). Similarly, two human 3 T-fMRI studies investigated whether FC-modulation in the resting-state was associated with a previous declarative learning task (Risius et al., 2018; Deantoni et al., 2021). Although these studies detected a few modulated connections in predefined regions activated during encoding, these were not correlated with memory performance and not controlled for the natural variation of the participant’s resting-state between consecutive measurements. In contrast, for a procedural memory task such as sequential finger-tapping, 3 T-fMRI did not detect a replay pattern during sleep (Rasch et al., 2007) or in the resting-state. Of note, sleep improved the movement speed of a motor task, but not its accuracy (Rasch et al., 2007; Ramanathan et al., 2015). Furthermore, analysis of the resting-state revealed enhanced connectivity in executive and cerebellar networks after motor learning but not after motor movement in and of itself (Jiang et al., 2004; Albert et al., 2009b). Therefore, whether it is possible to detect a replay response coupled to a preceding motor task remains unknown.

In this study, we set out to investigate the characteristics of 7 T-fMRI compared to 3 T-fMRI in the human brain beyond the well-described effect of increased SNR. For this purpose, we adjusted the SNR between both field strength through a targeted variation of the voxel size. As a result, no differences in spatial sensitivity of the functional response were found between the two field strengths. Therefore, we sought to examine whether 7 T offers a higher BOLD specificity for examining motor task-driven neuronal events affecting the connectivity in the subsequent resting-state. This connectivity modulation would be a functional correlate of the neuronal offline replay following a finger-tapping motor task.

## 2. Methods

### 2.1. Participants

A total of 18 (eight female, 10 male) healthy right-handed participants aged 19–55 years (average 37 years) were recruited. 11 participants had previous experience in being MRI scanned. Exclusion criteria included occurrence of any current or past form neurological/psychiatric diseases or having any contradictions to fMRI scanning. Ethical approval (189-15B) was provided by the local ethics committee of FAU, and informed consent was obtained from all participants. The study adhered to the tenets of the Declaration of Helsinki.

### 2.2. Study design

**Figure 1:**
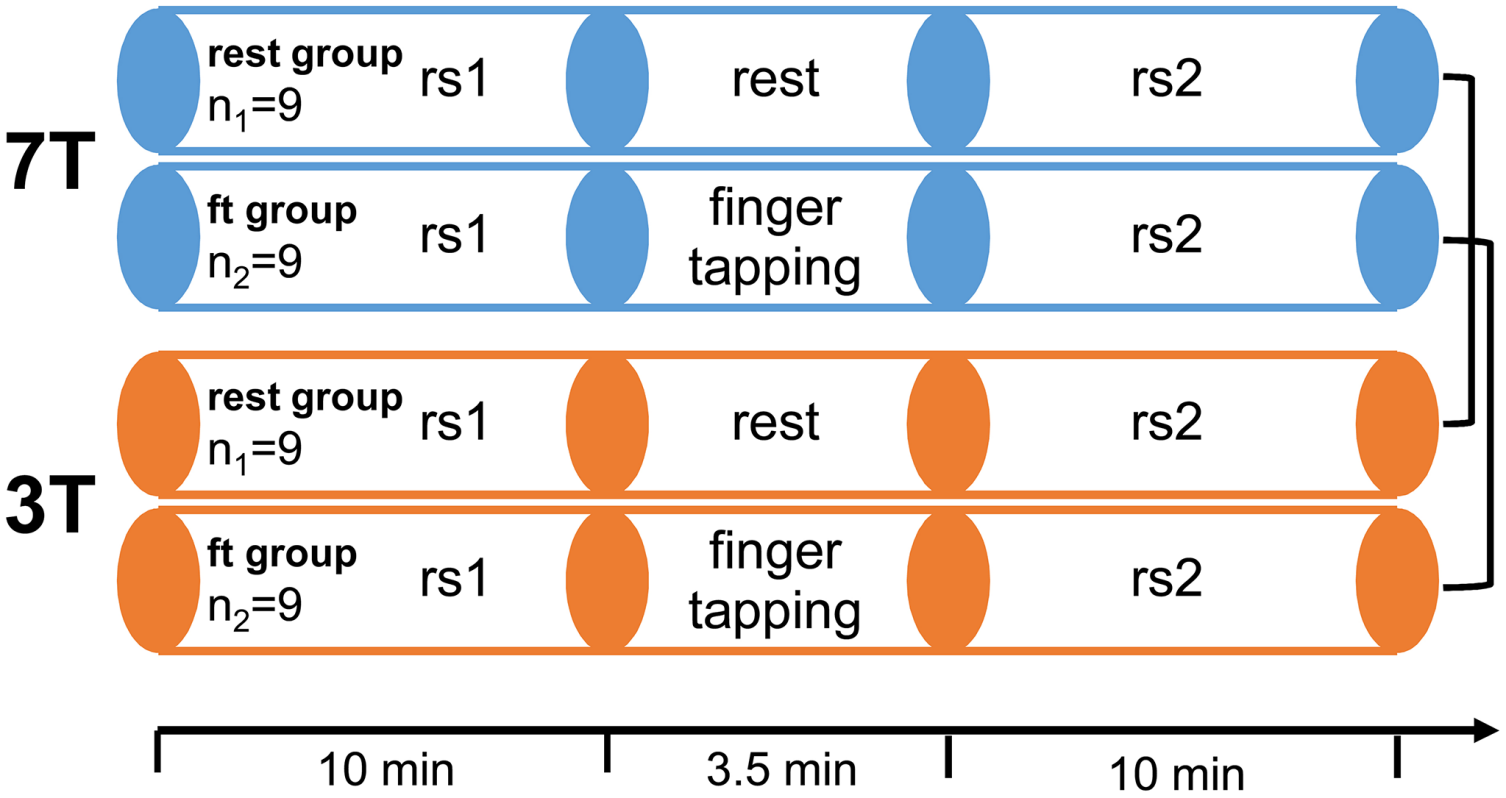
Experimental design. 18 healthy participants were measured twice, once in a 7 T and once in a 3 T Siemens Magnetom Scanner in a randomized order. Each session consisted of two resting state scans (rs1 and rs2) with either rest or a simple finger-tapping motor task in between. In the active motor task, participants tapped each finger sequentially for 14 s, followed by 14 s of rest, for a total of seven times after an initial 14 s rest (starting and stopping upon voice commands).

To compare the effects of high magnetic fields on functional MRI, we conducted a paired study design. Each participant underwent one 3 T and one 7 T measurement, respectively. The measurement order was balanced between all participants. The time between both measurements ranged from 1 to 6 weeks. Each fMRI session started and ended with resting-state measurements. During sessions, participants either remained at rest (rest-group) or executed an active right-hand finger-tapping motor task (ft-group). ft- and rest-group allocations were randomized under the constraint of balanced gender, age, measurement order, and previous MR experience (Figure 1).

### 2.3. fMRI stimulation paradigm

The motor stimulation was a right hand sequential tap of each digit with the thumb lasting 14 s. This finger-tapping stimulation was repeated seven times with baseline intervals of 14 s and additional 14 s baseline before the first finger-tapping. Thus, the duration of the whole stimulation sequence was 3 min 30 s. The participants were instructed to start and stop the finger-tapping by voice commands.

### 2.4. Acquisition

MRI scans were performed on Siemens Magnetoms TERRA (7 T) and TRIO (3 T) using 32 channel head coils. Prior to the functional scans an anatomical scan and a gre-field mapping was performed. The following setting were used for MP2RAGE at 7 T: TR = 4,500 ms, TE = 2.27 ms, GRAPPA 3, 0.8 mm isotropic resolution, TI = 3,200/1,000 ms, flip angle = 4°, TA = 9:15 min; and MPRAGE at 3 T: TR = 1.9 s, TE = 2.52 ms, TI = 900 ms, flip angle = 9°, 1 mm isotropic resolution, GRAPPA 2, TA = 4:26 min. Gre-field mapping was done at 7 T using a flash sequence (TR = 4.4 ms, TE = 1.02, 3.06 ms, 3.9 mm isotropic resolution, flip angle = 10°, GRAPPA 2, TA = 12 s) and at 3 T using the Siemens product sequence (TR = 650 ms, TE = 4.92, 7.38 ms, 2 mm isotropic resolution, flip angle = 60°, TA = 2:27 min). The anatomical scan was solely for clinical purposes on request of the participants. To acquire functional MRI scans, we used gradient echo-planar imaging sequences (GE-EPI) with following settings: GRAPPA (three with 48 reference lines), TR = 2 s, TE = 21.0 ms (7 T) and 29.6 ms (3 T), flip angle = 69° (7 T) and 73° (3 T), acquisition matrix = 168 × 168 (7 T) and 126 × 126 (3 T), no. of slices = 84 (7 T) and 72 (3 T), FOV = 252 mm × 252 mm, resolution = 1.5 mm^3^ (7 T) and 2 mm^3^ (3 T) isotrop. The resolution was adjusted to achieve comparable signal-to-noise ratios for 7 and 3 T: since it can be assumed that noise increases at least linear with B0 (Pohmann et al., 2016), the resolution ratio was chosen to match the inverse field strength ratio, i.e., 1.5^3^/2.0^3^ ~ 3/7. Both resting-state measurements acquired 300 volumes each (total time 10 min) and the sequence between both resting-state, either with or without finger-tapping, contained 105 volumes (total time 3 min 30 s).

### 2.5. Preprocessing of functional MRI data

After discarding the first two volumes to avoid MR saturation effects, fMRI data were distortion corrected using the acquired field map, corrected for slice scan time (cubic spline interpolation considering the scan order table) and motion (trilinear detection and sinc interpolation), and were subsequently smoothed spatially (3D Gaussian filter with FWHM 4 mm). The temporal dimension of the stimulus driven BOLD data was smoothed using a GLM-Fourier-Filter with 2 cycles and that of the resting-state data was band pass filtered with a frequency cut off between 0.009 and 0.08 Hz. All preprocessing steps except the band pass filtering of the resting-state data were performed using Brainvoyager QX (Brain Innovation, Maastricht, Netherlands; V2.8.2.2523). Band-pass filtering and all further analysis, if not stated otherwise, was done with MagnAn (Biocom GbR, Uttenreuth, Germany, V2.5), an IDL application (Exelis Visual Information Solutions Inc., a subsidiary of Harris Corporation, Melbourne, FL, United States, V8.5) designed for complex image processing and analysis with emphasis on (functional) MR imaging.

### 2.6. Individual brain atlas registration and brain region segmentation

To identify anatomical brain regions, we developed a modified Montreal Neurological Institute (MNI) probabilistic brain atlas that was a combination of the Harvard-Oxford-cortical-and-subcortical atlas including white matter and ventricles (Desikan et al., 2006), the fsl-oxford-thalamic-connectivity atlas (Behrens et al., 2003), and the SUIT cerebellum atlas (Diedrichsen et al., 2009). Additionally, a skilled neuroanatomist manually divided the brainstem into medulla, pons, left and right tegmentum, and left and right tectum. In total, this atlas spanned 166 brain regions plus white matter and ventricle regions. We used the first volume of each functional MRI sequence (with the skull stripped manually) as an anatomical reference, which we registered to the linear ICMB152 T2 template in MNI space using a diffeomorphic registration algorithm provided by Advanced Normalization Tools (ANTS; Avants et al., 2011; http://stnava.github.io/ANTs/). Subsequently, the resulting individual transformation matrices and fields were applied backward on each volume of the probabilistic atlas, i.e., each brain region. The median-filtered (kernel 3) maximum probability maps in the individual space of each participant were then used to define brain regions for further analysis. We focused on grey matter brain regions for resting-state data analysis, leaving white matter and ventricles as additional ROIs. Finger-tapping stimulation data were analyzed using brain regions covering both grey and white matter but excluding ventricles.

### 2.7. Quality metrics

The preprocessed first resting-state scan (rs1) was used to determine the following quality metrics:

Signal-to-noise ratio (SNR; Magnotta et al., 2006): The mean intensity within the gray matter divided by the standard deviation of the values outside the skull. Higher values are better.
Contrast-to-noise ratio (CNR; Magnotta et al., 2006): The difference of gray matter and white matter mean intensity values divided by the standard deviation of the values outside the skull. Higher values are better.
Foreground to background energy ratio (FBER; Shehzad et al., 2015): The variance of intensity values inside the brain divided by the variance of intensity values outside the skull. Higher values are better.
Entropy focus criterion (EFC; Atkinson et al., 1997): The Shannon entropy of volume voxel intensities proportional to the maximum possible entropy for a same sized volume. This quality metric indicates ghosting and head motion induced blurring. Lower values are better.
Temporal signal-to-noise ratio (tSNR; Murphy et al., 2007): Voxel wise calculated mean signal over time divided by the standard deviation over time, yielding tSNR volume maps. Higher values are better.
zDVARS (Afyouni and Nichols, 2018): DVARS is the standard deviation of the temporal derivative of the data, calculated as the spatial standard deviation of the temporal difference image. For better inter-cohort comparisons, DVARS was scaled relative to its temporal standard deviation and autocorrelation. Lower values are better.
Median distance index (MDI; Cox, 1996): The mean distance (1-spearman’s rho) between each time point’s volume and the median volume. Lower values are better.
Global correlation (Gcorr; Saad et al., 2013): The average correlation of all pairs of voxel time courses inside the brain indicating global data fluctuations. Values closer to 0 are better.

Grey matter and white matter regions were defined using the corresponding ROIs resulting from the individual brain atlas registration. Background regions were automatically defined as the largest contiguous region of all voxels outside the brain tissue mask with lower intensity than the 5% quantile within the brain mask. Thus, skull and muscles outside the brain tissue mask are reliably eliminated.

### 2.8. Analysis of finger-tapping stimulation data

Preprocessed finger-tapping stimulation data underwent a classical General Linear Model (GLM) analysis with the hemodynamic response function (HRF) convolved with the boxcar stimulation function as only predictor. This first step was done in Brainvoyager QX, further analysis of the resulting Statistical Parametric Maps (SPMs) was performed in MagnAn. To identify significantly activated voxels, the individual SPMs were thresholded using the Benjamini-Yekutieli version of the False Discovery Rate (Benjamini and Yekutieli, 2001; BY-FDR, *q* = 0.05, *n* = 748 time points) which considers a dependency between multiple tests. Since neighboring voxels influence each other this version seems to be the most appropriate to correct for voxel-wise statistical tests. Significantly activated voxels were assigned to distinct brain regions by multiplying the thresholded SPMs with the corresponding maximum probability map. The proportion of participants per group that showed any activated voxels within a certain brain region was defined as the activation probability of that brain region. Under consideration of the different voxel resolutions for 7 T and 3 T measurements, the number of activated voxels was expressed as activated volumes (mm^3^) per brain region and measurement. For further analysis only those regions were taken into account which exceeded an activation volume larger than 15 mm^3^ and had, under this constraint, an activation probability of 100% either in the 7 T or in the 3 T group (in total 43 regions, including bilateral counterparts, see Supplementary Table 1). The average time course of activated voxels within these brain regions was extracted and all stimulation periods (“ON” in the boxcar function) including five baseline time points (“OFF” in the boxcar function) before and after the stimulation period were averaged. BOLD response was expressed as percent BOLD signal change (Δ*R*/*R*) using the average of the three inner time points of the baseline before the stimulation period as baseline reference *R*. Using this BOLD response amplitude, the following parameters were calculated to characterize the stimulation response: amplitude (peak) height (PH), time to peak (PT), amplitude width (PW), and amplitude symmetry (PS). For detailed description, see Supplementary Figure 1.

Additionally to those regional parameters we calculated the temporal contrast-to-noise ratio tCNR (Geissler et al., 2007) per activated voxel. tCNR was calculated from ΔS_CNR_/σ_t-noise_, with ΔS_CNR_ defined as the following difference:

$\begin{array}{l} \left(\text{Mean value of all}\;{\text{time points within the stimulation period}}^{“}{\text{ON}}^{”}\right)\\ -\left(\text{mean value of all}\;{\text{time points within the baseline period}}^{“}{\text{OFF}}^{”}\right) \end{array}$

σ_t-noise_ is the standard deviation of the difference between the original and smoothed signals indicating the non-task-related variability over time. Smoothing was performed using a Savitzky–Golay filter with a polynomial order of 2 and length 5. Voxel-wise tCNR was averaged over activated voxels for each brain region.

### 2.9. ICA analysis of resting-state data and identification of resting-state networks

Prior to further resting-state analysis, the average time courses of white matter and ventricles was regressed out of the preprocessed and band pass filtered resting-state data. White matter and ventricle masks were defined from the individual brain region segmentation as described above.

For ICA analysis, the whole rs1 scans of all participants were registered to the T2 MNI template by repetitively applying the transformation matrices calculated for the individual brain atlas registration. Group ICA analysis of concatenated time series was performed separately for 7 and 3 T scans using the FastICA algorithm (Calhoun et al., 2001) in the “Group ICA of fMRI Toolbox” (GIFT v1.3g; https://trendscenter.org/software/). 20 independent components were calculated. For group comparison (7 vs. 3 T) only the aggregate components were used.

We identified common resting-state networks (RSN) by comparing the 20 GIFT 3 T group ICA aggregate components with two different public available template sets provided in the MNI152 space. The first template,^1^ published by Smith et al. (2009), is based on images of 36 healthy subjects, who were scanned with a 3 T Siemens TRIO Magnetom and analyzed using GIFT group ICA with 20 components. Except for the length of the measurement (6 min instead 10 min in our study), this was the same protocol as we used. Therefore, this template (further on called Smith Template) was most suitable to serve as a reference for the identification of RSNs within our datasets. The second template was provided by the Stanford University and is referred to as Stanford Template.^2^ The authors (Shirer et al., 2012) measured 15 subjects for 10 min using a 3 T GE Scanner and subsequently calculated a MELODIC group ICA with 30 components. 14 RSNS were identified by visual inspection and were arbitrarily binarized to obtain in total 90 distinct functional ROIs. Two inconsistencies between both templates had to be solved: (1) Due to the higher number of ICA components to create the Stanford template, the DMN was separated into a ventral and a dorsal part. Those two RSNs were combined to one. (2) The Smith Template contained three networks, named executive control and left and right frontoparietal networks, which visually match the anterior Saliency and left and right executive control network of the Stanford Template. Those matching RSNs were considered as corresponding templates and named according to the Stanford template anterior Saliency (aSN, matching the executive control of the Smith template), left and right executive control (LECN and RECN, respectively, matching the left and right frontoparietal network of the Smith template).

Similarity between Smith templates and 3 T ICA components was assessed by spatial correlation of the *z* score values of all voxels within the brain. Stanford templates were compared with the binarized ICA aggregate components (*z* score > 2, corresponding to *p* < 0.05, uncorrected) by spatial overlap with reference to the template and additionally the similarity was determined using the Jacquard index. RSNs were automatically identified by the maximum similarity converging in both directions (best match of all ICA components to a specific template and best match of all templates to specific ICA component) for either the Smith or the Stanford template. Subsequently, the 7 T RSNs were identified by spatial cross correlation of the 20 7 T ICA components to the previously defined 3 T RSNs. All automatically identified RSNs were confirmed by visual inspection.

### 2.10. Graph-theoretical resting-state analysis using multi seed correlation

According to the ICA analysis, the residuals of the preprocessed data after regression of white matter and ventricle time courses were used. Graph-theoretical resting-state analysis describes brain regions as nodes and focusses on the connections between each pair of brain region, called edges in network terminology. To assess the functional connectivity between the brain regions we used a multi-seed-region approach (MSRA) introduced by Kreitz et al. (2018). Briefly, a predefined seed region was placed automatically in the center of mass of each brain region as determined by atlas registration and region segmentation described above. Seed regions were spheres with approximately 7.5 mm diameter. Due to the different voxel resolution of 7 and 3 T measurements odd kernel sizes to achieve closest diameters were five voxel for 7 T (7.5 mm diameter) and three voxel for 3 T (6 mm diameter). The average time course of each seed region was correlated with every voxel time course within the brain and the resulting correlation maps were thresholded using BY-FDR (*q* = 0.05, *n* = 298 time points) to determine significantly correlating voxels. In opposite to the predefined seed regions the location of those target voxels per brain region was determined purely data driven, thereby enhancing sensitivity of the connectivity between pairs of nodes (Kreitz et al., 2018). The average Pearson’s correlation *r* of all target voxels per brain region was used to define the connectivity strength to the respective seed region. This procedure was repeated for every brain region resulting in an asymmetric correlation matrix per resting-state scan. For further analysis, Pearson’s *r* values were transformed to Fisher’s *z*-values to provide normal distribution.

### 2.11. Resting-state quality measures

Quality of resting-state connectivity was assessed via the reasonable assumption that homotopic brain regions in healthy subjects are stronger connected than heterotopic, independently of their anatomical distance (Salvador et al., 2005). For each bilateral brain region, the normalized rank of the connectivity strength to its counterpart in the opposite hemisphere within all its connections was determined. Subsequently, a linear fit over the ranks of all brain regions in dependence on the anatomical distance was calculated. Group comparisons between 7 and 3 T resting-state matrices were conducted with the fitted rank for the mean anatomical distance and the slope of the linear fit. The first indicates the general dominance of bilateral interhemispheric connectivity and the latter the dependency on anatomical distance.

Another measure for resting-state data quality is the specificity for distinct RSNs. In theory, two core regions of the default mode network should correlate stronger than on of these regions to a core region of another, preferably task positive network (Grandjean et al., 2020). Here, we calculated the correlation ratio of the anterior cingulate cortex to the middle cingulum (part of the central axis of the default mode network) and to the middle frontal gyrus (core region of the executive control network). Correlation of all hemispherical combinations were averaged (left to left, right to right, left to right, and right to left). Higher values indicate higher specificity.

### 2.12. Topological comparison of resting-state graphs

For topological comparison, the 7% strongest connections of the average matrices per group (7 and 3 T) were extracted to create average networks of the same density. Network communities were detected using a heuristic method that is based on modularity optimization proposed by Blondel et al. (2008). The nodes within these communities are more strongly connected to each other than to nodes outside the community. Networks were visualized in AMIRA (Thermo Fisher Scientific Inc., Waltham, MA, United States, V5.4.2) using a force-based algorithm (Kamada and Kawai, 1989).

Topological components that represent subnetworks of altered connectivity strength between the 7 T rs1 and the 3 T rs1 scan were determined using an adaptation of the network-based statistics (NBS; Zalesky et al., 2010). NBS is a method to control the family-wise error rate after mass univariate *t*-tests performed at every single network edge. NBS exploits the interconnected extent of univariate significant different edges by permutation of subject specific networks between experimental groups. In a paired design, we introduced an additional paired control group in order to control for general effects of repeated measurements (pNBS; Kreitz et al., 2018). Here, we used an unpublished control data set of 11 healthy subjects (seven females, age 25–63) who were measured twice with an interval of 2 days on the same Siemens TRIO Magnetom Scanner (GE-EPI, TR: 3 s, TE: 30 ms, flip angle: 90°, matrix size 128 × 128 pixel, pixel resolution: 1.5 mm × 1.5 mm, 36 slices, slice thickness: 3 mm, slice gap: 0.75 mm, 200 volumes). Preprocessing and MSRA analysis were performed as described above. This control group was used to define an α-value where almost no significantly different connections between the repeated measurements occurred. To minimize the effect of single outliers, we calculated the 99% quantile of the mass univariate paired *t*-statistics *p* values. The resulting α-value was used to identify a set of supra-threshold connections (control component). The same threshold was applied to the paired *t*-statistics *p* values of the experimental group (i.e., paired rs1 scans with 7 and 3 T), and all remaining connected components equal or smaller to the control component were eliminated. Finally, the family-wise-error (*p*_FWE_) was controlled by 10,000 randomized permutations of pairs between experimental and control group (for details, see Kreitz et al., 2018). The α-value ensures that the observed differences correspond dominantly to the experimental conditions, in this case the field strength, whereas the p_FWE_ value indicates the probability that the observed component is not random. NBS and pNBS are weak controls, which only allows rejecting the global null hypothesis. Thus, no single connections but rather the whole component mirrors the resting-state modulations under the experimental conditions.

### 2.13. Variability and reproducibility of resting-state correlation matrices

Variability of resting-state graphs between subjects was assessed via pairwise spatial correlation of the underlying MSRA matrices, resulting in a correlation matrix which represents the similarity of each subject with all other subjects. Reproducibility of two subsequent resting-state measurements was determined by spatial correlation of the MSRA matrices of rs1 and rs2 separately for each subject. Here, only subjects without finger-tapping stimulation between both resting-state measurements were taken into account. Correlation values were transformed into Fisher’s *z*-values.

### 2.14. Analysis of resting-state modulations

Since we expect only tiny modulations in distinct brain regions, pNBS was not powerful enough to detect more modulations between rs1 and rs2 in the finger-tapping group compared to the rest-group. However, reinforced by the observed reduced variability between subjects in rs2 (see the section 3), the classical NBS (α = 0.05, 10,000 permutations) had enough power to detect significant components in rs2 that distinguish between groups. This first step of the resting-state modulation analysis was completely data driven and was applied to rs1 and rs2, respectively.

The dominant regions within the significant component resulting from the rs2 group comparison, i.e., those with the most modulated connections, were used for further analysis. This second step should identify specific connections that are significantly modulated due to the finger-tapping task performed between both resting-state measurements. For this, we used the region specific seed correlation maps (SCM) calculated during the MSRA procedure for rs1 and rs2 in both groups. The MSRA algorithm ensured that the seed regions were subject- and measurement-specific placed according to their relative position within the brain region of interest and sized. Only voxels with significant correlation of their time courses to the seed region time course as identified using BY-FDR were taken into account. SCMs of the selected regions were registered to the MNI space by applying the transformation resulting from the diffeomorphic registration of their corresponding anatomical references (see the section “2.6”).

Subsequently, a voxel-wise paired *t*-test between rs1 and rs2 was performed separately for each group. The resulting statistical *t*-maps was cluster enhanced using threshold-free cluster enhancement (TFCE with parameter settings signal height H = 2 and cluster extent E = 0.05; Smith and Nichols, 2009) and corrected for multiple comparison using permutation testing. Relying on the assumption that significance of a contiguous cluster is more likely to be true positive than that of a single voxel, TFCE aims to enhance areas of *t*-values that exhibit some spatial contiguity without the need for a hard cluster-forming thresholding. The resulting modified values are normalized back to the range of the original *t*-values. Randomized permutation testing was performed with 1,000 repetitions. To avoid outliers and therefore improve statistical power the 99.9th percentile of all voxel *t*-values within the brain instead of the maximum *t*-value was used to create the “Null”-distribution. The 95% quantile of this distribution represented the rejection condition and the normalized TFCE image was thresholded with this *t*-value to obtain significant clusters. The combination of both methods provided enhanced sensitivity (less false negative, reduced Type II error) of statistical tests on behalf of acceptable costs in specificity (more false positive, enhanced Type I error). Binarized significant clusters of the ft- and the rest-group were combined by a logical OR. This resulted in a binary mask that marked all voxels that are significant in either of both groups. This mask was applied to each subject’s rs2–rs1 difference map and, after brain region segmentation using the maximum probability map of the above-described 4D probability atlas in MNI space, the average difference per subject and brain region was extracted.

To obtain specific connectivity modulations due to finger-tapping, the rest-group serves as a control and region specific average rs2-rs1 differences were tested for significance between ft- and rest-group using a homoscedastic *t*-test and Benjamini-Hochberg FDR (Benjamini and Hochberg, 1995; BH-FDR, *q* = 0.05) to correct for multiple comparison. The resulting connections were visualized as network graphs with brain regions as nodes and the modified connections of the seed regions as edges using AMIRA (Thermo Fisher Scientific Inc., Waltham, MA, United States, V5.4.2).

### 2.15. Statistics

In addition to network based statistics (NBS and pNBS) and voxel wise paired *t*-test including TFCE, as described above, the following statistical approaches were used:

Principle Component Analysis was performed to separate 3 and 7 T measurements by quality metrics (*n* = 18) and by BOLD response parameters including tCNR (*n* = 9). To search for group specific BOLD response parameter effects, we performed a mixed repeated measure ANOVA with replication using field strength as within factor and brain regions as between factor (*n* = 9). Group effects of the activation probability were assessed using two factor ANOVA without replication with factors field strength and brain regions. Variability main effects between measurements were examined by one factor repeated measure ANOVA using Fisher’s *z* correlation values of all subject pairs as a measure of similarity (*n* = 36). Significant ANOVA effects and interactions were then tested using Tukey HSD. Direct two group comparisons were made using Student’s *t*-test, either paired (3 vs. 7 T, rs1 vs. rs2) or homoscedastic (ft vs. rest). Multiple tests were appropriately corrected using permutation tests (NBS, pNBS, voxel wise *t*-statistic, description see above), Benjamini-Yekutieli FDR (*q* = 0.05, detection of significant BOLD response and resting-state correlation in volumes with voxel wise statistic), Benjamini-Hochberg FDR (*q* = 0.05, detection of significant group differences in region specific statistics) and Bonferroni (correction of ANOVA follow up Tukey HSD statistics). Significance level was *p* < 0.05.

## 3. Results

### 3.1. 7 and 3 T functional data can be differentiated mainly by spatial quality metrics

To evaluate the measurement quality for each session, we assessed the first resting-state rs1 across all participants for spatial (average brain volume over time) and temporal (voxel-wise time-courses) quality metrics. To evaluate the spatial data quality, we determined the SNR, the CNR between grey and white matter, the foreground to background energy ratio (FBER), and the entropy focus criterion (EFC). Conversely, we assessed temporal data quality using the temporal SNR (tSNR), the standardized per-image standard deviation of the temporal derivative (zDVARS), the median distance index (MDI), and finally the global correlation (Gcorr). For details, see the section 2.

**Figure 2:**
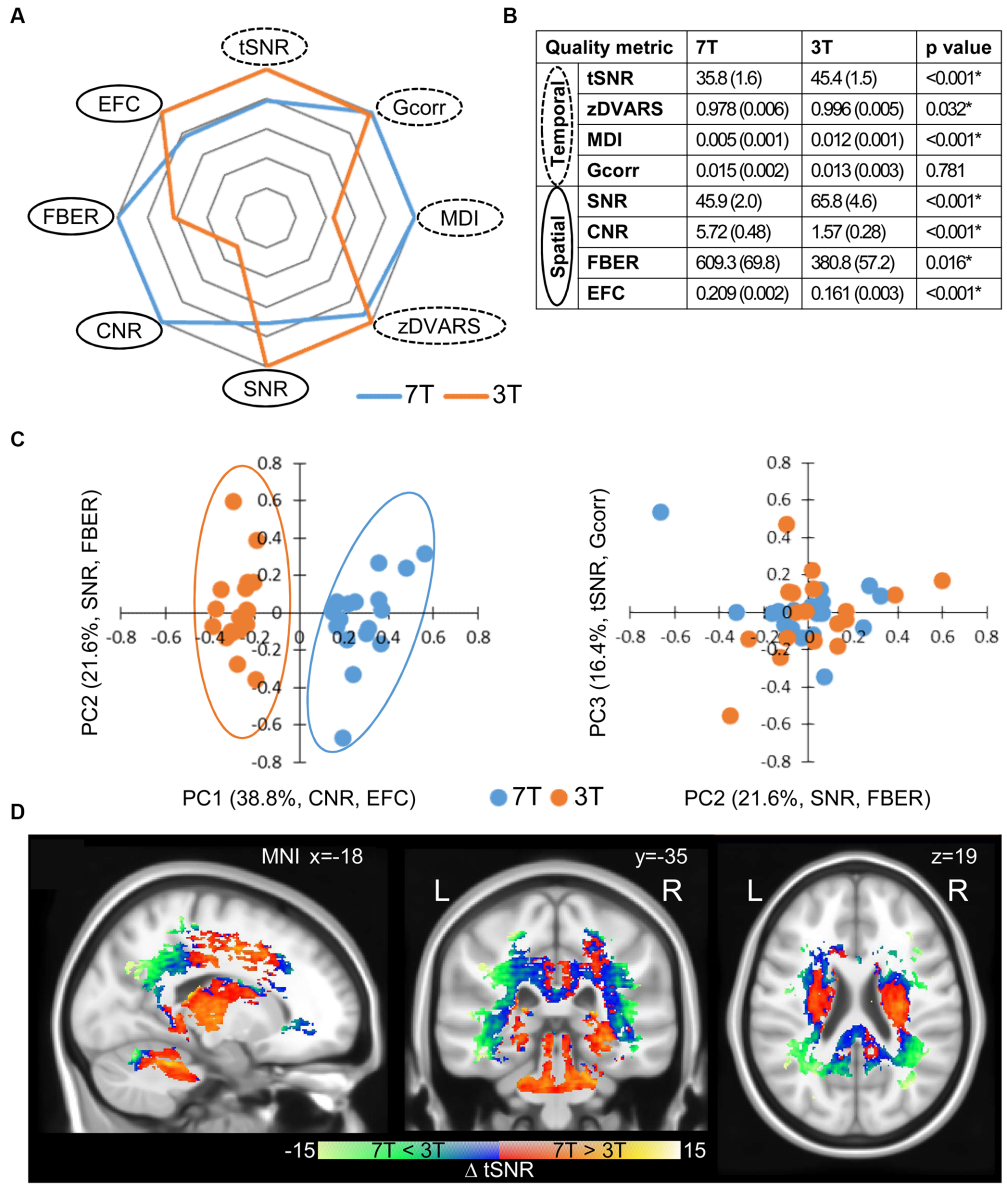
Quality metrics of the first resting-state scan rs1. **(A)** Fingerprints of average quality metrics for 7 and 3 T (solid line spatial, dashed line temporal metric). The better value of 7 or 3 T is set to 1.0 and the corresponding one is given proportionally. **(B)** Mean quality measures with standard deviation in brackets (*n* = 18). Significance was determined by paired two tailed *t*-test (^*^p < 0.05). **(C)** Principle component analysis (PCA) of quality metrics. Projections of single subject measurements on the first and second PC (left) and the second and third PC (right). Percent eigenvalue and metrics with absolute loadings above 0.5 on the respective PC are given in brackets of axis titles. For detailed loadings of the PCs, see Table 1. **(D)** Spatial distribution of significant differences in tSNR between 7 and 3 T (*n* = 18, two tailed paired *t*-test with permutation correction, *p* < 0.05).

Overall, those metrics generated high quality fingerprints (Figure 2A) that, with the exception of Gcorr, differed significantly between 7 and 3 T (Figures 2A,B, paired *t*-test, *p* < 0.05). When we did a principal component (PC) analysis using all metrics, we found an excellent separation of field strengths along the first PC (Figure 2C) with highest absolute loadings for CNR and EFC in the spatial dimension. Both measures are indicative of the technical quality of the MR measurements, but have no influence on the functional data processing. EFC is a measure of image blur due to ghosting and head motion. Since DVARS, another measure of motion artefacts, did not differ between the 3 and 7 T measurements, it is very likely that the higher EFC of the 7 T measurements is caused by increased ghosting. The higher CNR at 7 T is a measure of the improved performance of the 7 T scanner, which is not directly related to SNR and voxel resolution. In contrast, temporal metrics did not contribute before the third PC and did not account for field-strength dependent group separation (Figure 2C; Table 1), which means that, under the constraint of adjusted resolutions to compensate for field strength-dependent SNRs, temporal data quality is less influenced by higher field strength than spatial image quality. We found that whole brain average SNR and tSNR were even higher for 3 T. As expected, the highest tSNR of the 3 T measurements was located within the cortex, which was closer to the f-MRI head coil. In contrast, the tSNR of 7 T measurements was instead more evenly distributed throughout the brain (Supplementary Figure 2). Taken together, the higher cortical tSNR at 3 T did not survive the correction for multiple comparison, whereas subcortical regions and the cerebellum showed a significantly higher tSNR at 7 T (Figure 2D, paired *t*-test, corrected, *p* < 0.05).

**Table 1:** PCA of quality metrics.

Eigenvalue (%)		38.8	60.4	76.8	87.9	95.9
Principle component		PC1	PC2	PC3	PC4	PC5
Temporal	tSNR	−0.288	0.258	−0.572	−0.087	0.479
	zDVARS	−0.212	0.449	−0.148	−0.441	−0.691
	MDI	−0.376	−0.271	0.451	0.268	−0.235
	Gcorr	0.067	−0.100	−0.566	0.704	−0.402
Spatial	SNR	−0.272	0.557	0.279	0.330	0.082
	CNR	0.509	0.195	0.030	−0.041	−0.214
	FBER	0.304	0.543	0.219	0.332	0.150
	EFC	0.548	−0.070	0.011	−0.100	0.029

Loadings of the first five principle components (PC). Loadings above 0.5 for each PC are highlighted in grey. Only PC1 contriute to the separation between 7 T and 3 T measurements.

### 3.2. Motor task activation patterns were consistent between field strengths, but temporal responses varied

**Figure 3:**
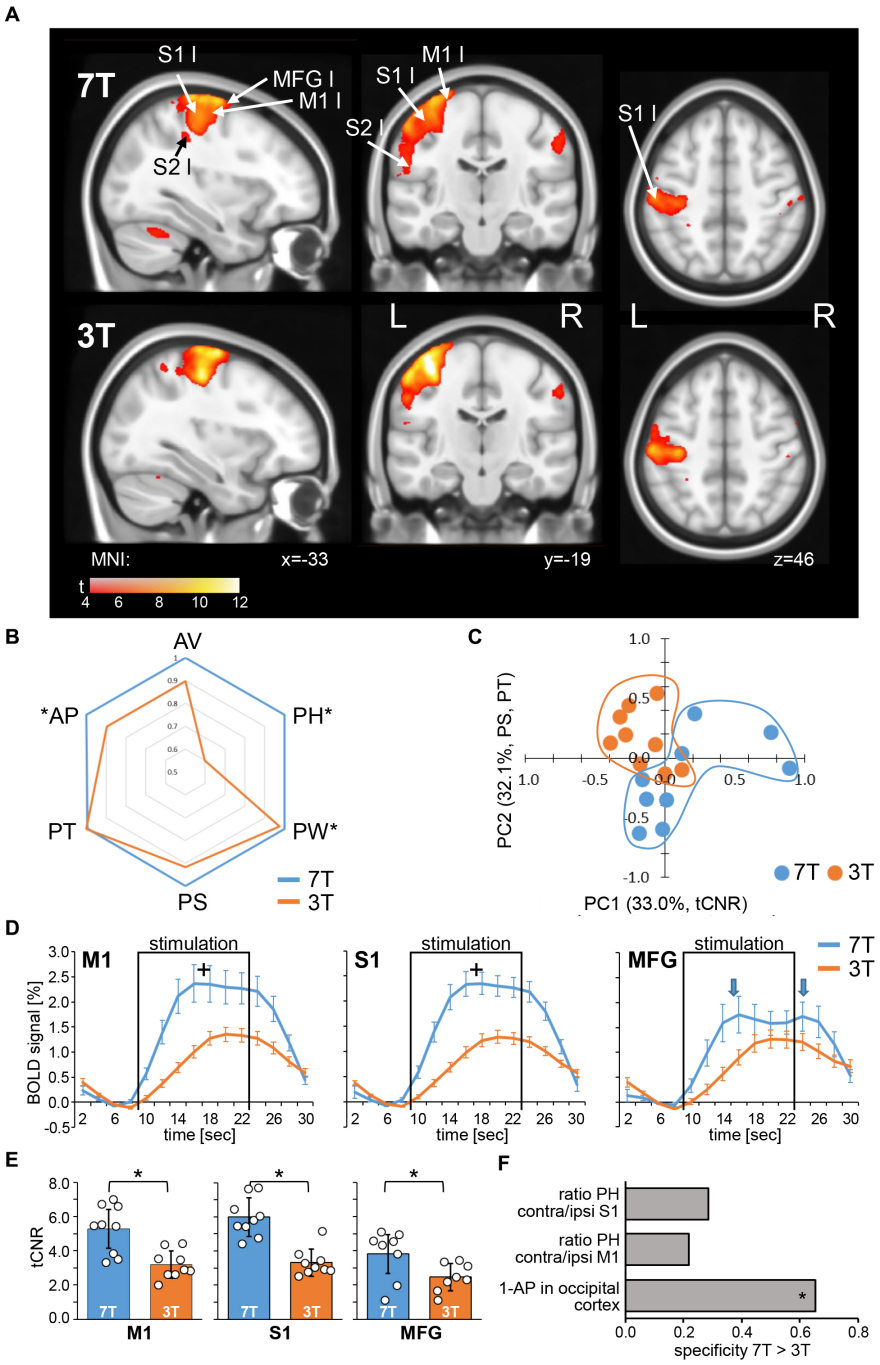
BOLD response to a finger-tapping motor task. **(A)** Neurological view of average statistical parametric maps, thresholded for significant activation using false discovery rate according to Benjamini and Yekutieli (2001) (BY-FDR, *q* = 0.05, *n* = 748 time points), acquired for each subject with 7 T (top) and 3 T (bottom), respectively (*n* = 9). Centroid MNI coordinates of activated areas per region are given in Supplementary Table 2. **(B)** Fingerprint plot (left) of normalized average BOLD response parameters (*n* = 9). Significance between 7 and 3 T was determined by mixed repeated measure ANOVA without replication for AP and with replication for all other parameters using field strength as within factor and brain regions as between factor (^*^p < 0.05, Bonferroni corrected). **(C)** PCA of average BOLD response parameters per subject (*n* = 9). Projections of single subjects on the first and second PC are shown. Percent eigenvalue and the BOLD response parameters with absolute loading above 0.5 on the respective PC are given in brackets in axis titles. For detailed loadings of the PCs, see Table 2. **(D)** Average BOLD response profiles of left primary motor cortex (M1), primary somatosensory cortex (S1), and middle frontal gyrus (MFG) and **(E)** the corresponding temporal contrast-to-noise ratios (tCNR). Squares mark the stimulation time period. Arrows indicate biphasic amplitude shape. ^+^*p* < 0.05, Tukey HSD with Bonferroni correction, ^*^p < 0.05, two tailed paired t-test with Bonferroni correction. **(F)** Specificity measures for 7 T in relation to 3 T task-related fMRI. ^*^p < 0.05, mixed repeated measure ANOVA without replication. AP, activation probability; AV, activated volume; PH, amplitude (peak) height; PS, amplitude symmetry; PT, time to peak; and PW, amplitude width (see Supplementary Figure 3).

Next, we evaluated how field strength influences a BOLD response stimulated by a motor task (*n* = 9, paired design). As an indicator for the region’s activation sensitivity and specificity, we expressed the motor task-induced activation per brain region as percentage of participants, the so-called activation probability (Supplementary Figure 3). In further analysis, we only included regions in which the activation probability was 100% in at least one hemisphere and with one field strength. The resulting 43 brain regions (21 bilateral and 1 middle) and their corresponding activation probabilities are shown in Supplementary Table 1. Within this motor response pattern, the average activation probability was significantly higher with 7 T compared to 3 T (two factor ANOVA without replication, *F* = 2.441, *p* = 0.002), indicating a higher functional sensitivity at 7 T. To assess the functional specificity, we selected a set of 10 occipital cortical regions that are very unlikely to be involved in motor responses and calculated the average activation probability (AP) of these regions to define a false positive rate. The inverse false positive rate, here 1-AP, is a measure of specificity. In relation to 3 T (1-AP = 37%), functional specificity at 7 T (1-AP = 64%) was significantly enhanced by 73% (two factor ANOVA without replication, *F* = 11.055, *p* = 0.012, Figure 3F).

Although spatial quality was higher for 7 T, we found highly similar spatial distributions of activation patterns for 7 and 3 T, especially for contralateral sensorimotor areas (Figure 3A, see also centroid distances of activated voxels in Supplementary Table 2). However, the average BOLD signal response profile revealed significantly higher (*F* = 334.955, *p* = 8.0 × 10^−59^, Bonferroni corrected, repeated measure ANOVA) and broader (*F* = 7.048, *p* = 0.040) response amplitudes with a tendency toward a longer decreasing phase (higher amplitude symmetry), but no difference in time to peak after stimulation onset (Figure 3B; Supplementary Table 1). Additionally, we calculated the temporal contrast-to-noise ratio (tCNR) per region and found a significant main effect for field strength (7 > 3 T, *F* = 489.46, *p* = 0.000). To identify parameters that contribute the most to a field strength-dependent measurement separation, we performed a PCA using all BOLD response parameters and the tCNR, each averaged per subject over all brain regions. Subject’s measurements were separated according to field strength along the first and the second PC. The first two PCs explained 65% of the variability between all subjects and loadings above 0.5 were tCNR on the first, and amplitude symmetry and time to peak on the second PC (Figure 3C). However, we found that the activated volume did not contribute to the data variability or separation between measurements according to field strength (Table 2).

**Table 2:** PCA of BOLD response parameters.

Eigenvalue (%)	33.0	65.0	86.0	96.7
Principle component	PC1	PC2	PC3	PC4
Activation volume	0.295	−0.115	−0.635	−0.674
Amplitude height	0.430	−0.479	0.318	0.172
Amplitude width	0.085	−0.281	−0.655	0.676
Amplitude symmetry	−0.448	−0.529	−0.065	−0.106
Time to peak	0.466	0.505	−0.104	0.185
tCNR	0.551	−0.379	0.228	−0.119

Loadings of the first four PCs. Loadings above 0.5 for each PC are highlighted in grey. Only PC1 and PC2 contribute to the separation between 7 and 3 T measurements.

When we investigated region-specific effects of field strength on brain regions using ANOVA, we did not find any interactions except for amplitude height (*F* = 2.009, *p* = 0.0004) and tCNR (*F* = 3.081, *p* = 1.47 × 10^−8^). Here, the contralateral primary motor (M1 left) and somatosensory (S1 left) regions showed significantly enhanced response amplitudes (Tukey HSD, corrected, *p* < 0.05; Figure 3D). To verify, that this observation indicate an enhanced specificity of the BOLD response, we compared the ratios of the contralateral response amplitude (specific response to the right hand motor stimulation) to their ipsilateral counterpart (non-specific response). For both regions, we found enhanced ratios with 7 T compared to 3 T fMRI, again indicating higher functional specificity at 7 T (Figure 3F). Furthermore, the tCNR was enhanced in the left S1 and M1 regions (Figure 3E), and additionally in bilateral secondary motor cortex and the deep nuclei of the cerebellum (data not shown, Tukey HSD, corrected, *p* < 0.05).

### 3.3. 7T resting-state networks showed higher specificity explicitly in cognitive and sensorimotor networks

To analyze resting-state data, we applied GIFT, a MATLAB tool for performing independent component analysis (ICA) on fMRI data, on the rs1 period (*n* = 18, paired design) for both field strengths. Specifically, we checked for common resting-state networks (RSN) and differences in spatial distribution and network level functional connectivity strength between 7 and 3 T. To identify RSNs, we compared the similarity of 20 ICA aggregate components derived from the GIFT group ICA to two sets of publicly available templates (Smith et al., 2009; Shirer et al., 2012; Supplementary Figure 4, Supplementary Results).

**Figure 4:**
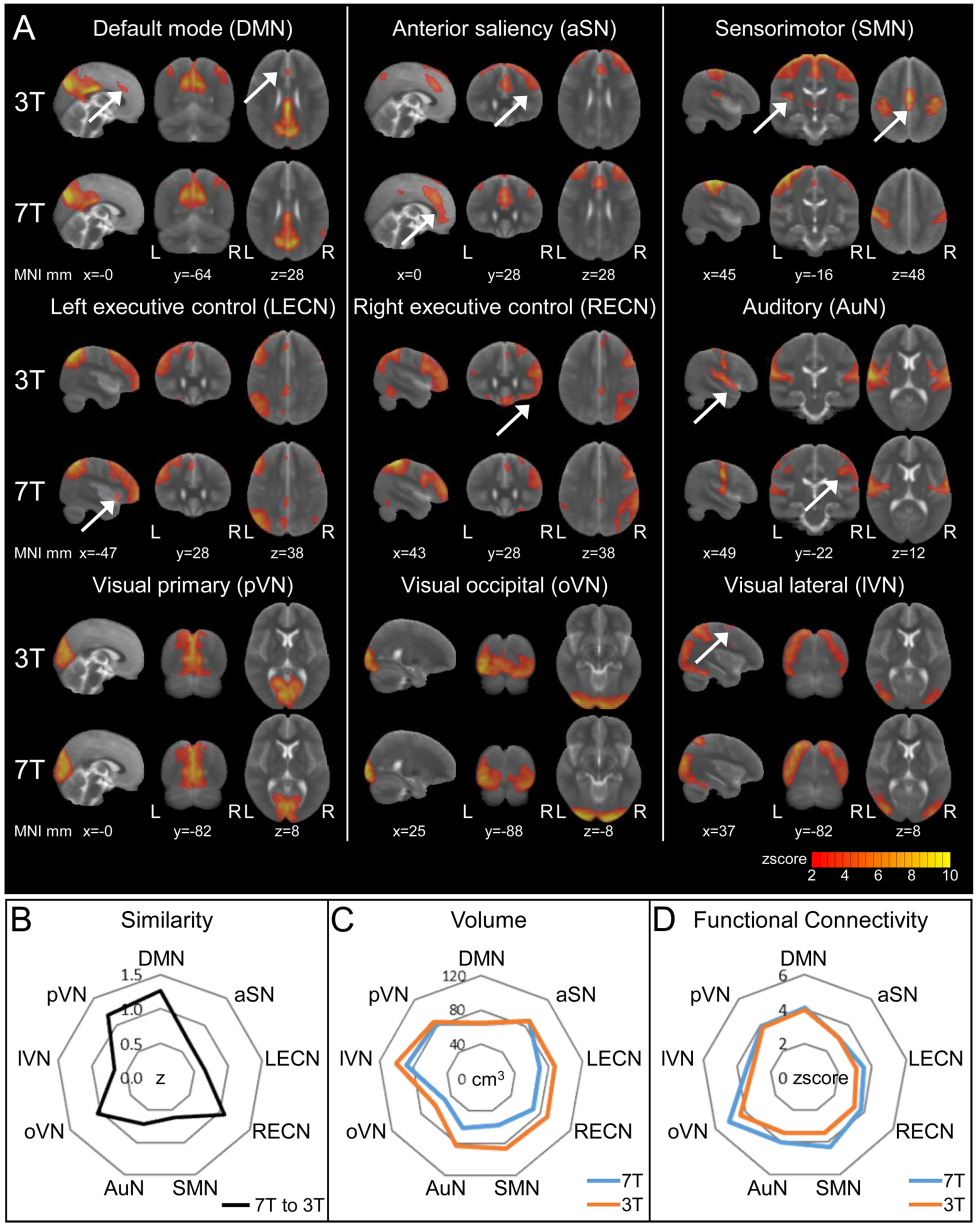
Resting-state Networks (RSNs) derived from 3 to 7 T fMRI and 20-component ICA. RSNs were identified in comparison to two published templates (see Supplementary Results and Supplementary Figure 2). **(A)** ICA *z* score maps thresholded at *z* score = 2 (corresponding to *p* < 0.05, uncorrected). The arrows indicate additional regions in either the 3T or 7T ICA component of the respective RSN. Visualization of the three most informative orthogonal slices for each 3/7 T pair superimposed on the MNI standard space template image. **(B)** Similarity of 3 and 7 T paired RSNs calculated via spatial cross correlation of ICA *z* score maps. **(C,D)** Comparison of thresholded ICA maps (*z* score > 2) of 3/7 T pairs using total volume **(C)** and average *z* score as a measure for functional connectivity **(D)**.

In all, we detected nine RSNs as follows: the default mode (DMN), anterior Saliency (aSN), sensorimotor (SMN), left and right executive control (LECN and RECN, respectively), auditory (AuN), and three visual networks: medial or primary (pVN), lateral (lVN), and occipital (oVN; Figure 4A). When we cross-correlated the corresponding ICA components to assess the similarity of the RSNs between 7 and 3 T, we found that the similarity was highest for the DMN (*r* = 0.85) and lowest for the SMN (*r* = 0.54) with a median value for all nine RSNs of *r* = 0.611 (Figure 4B). Next, we determined the global spatial extension of the thresholded RSN aggregate components (*z* score > 2, corresponding to *p* < 0.05, uncorrected) and the average *z* score as an indicator for network level FC strength. We found that spatial extension and FC were identical at 7 and 3 T for DMN, pVN, and aSN (Figures 4C,D). Those networks, especially the DMN, are considered task free networks associated with the basic function of the resting brain. However, the more cognitive executive control (LECN, RECN) and higher visual (lVN, oVN) networks and the SMN showed smaller spatial extension and enhanced average FC at 7 T (Figures 4C,D), that might indicate higher spatial specificity of these RSNs at 7 T.

### 3.4. 7 T resting-state graphs showed higher specificity of the underlying functional connectivity

Compared to the ICA-derived RSNs, graph-theoretical FC analysis generates deeper insights into the brain-wide information flow. For this purpose, we used the multi-seed-region approach MSRA (Kreitz et al., 2018) to create subject specific correlation matrices for rs1 using 158 brain regions (*n* = 18, paired design).

**Figure 5:**
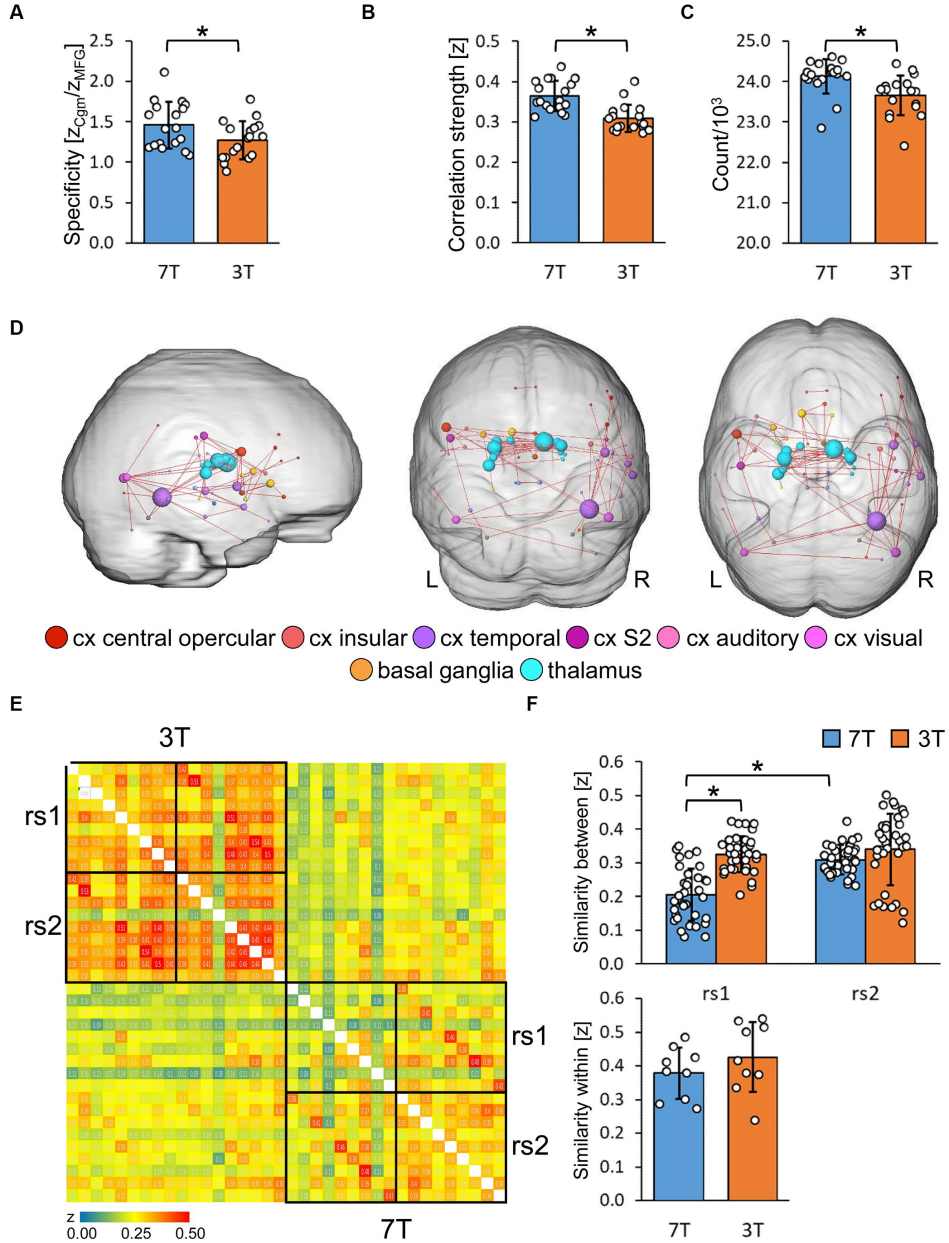
Evaluation of resting-state graphs derived from multi-seed-region analysis. **(A)** Specificity of resting-state graphs of the first scan (rs1) described as ratio of the functional connectivity strength (*z*) of the anterior cingulum to the middle cingulum (Cgm) and to the middle frontal gyrus (MFG; *n* = 18). Significance was determined by two tailed paired *t*-test (^*^p < 0.05). **(B)** Properties of rs1 graphs thresholded for significantly correlating connections via BY-FDR (*q* = 0.05, *n* = 298 time points). Left: average functional connectivity strength. Right: number of significant functional connections (*n* = 18). Significance was determined by two tailed paired *t*-test (^*^p < 0.05). **(C)** Significantly enhanced functional connectivity of 7 T compared to 3 T rs1 graphs with a connection density of 7% (*n* = 18). Only connections are shown that remain significantly strengthened after each measurement’s normalization to its average functional connectivity strength. Significant components were determined by paired controlled network based statistics (pNBS, α = 0.014, *p*_FWE_ < 0.0001). **(D,E)** Reproducibility and variability of both resting-state scans rs1 and rs2 at 3 and 7 T. Only participants with rest in between both scans were considered (*n* = 9). **(D)** Similarity matrix of all resting-state graphs assessed via spatial cross correlation of the MSRA *z*-correlation matrices. **(E)** Subject variability derived from pairwise correlation values. Top: between subject variability (*n* = 36 subject pairs, one factor repeated measure ANOVA followed by Tukey HSD with Bonferroni correction, ^*^p < 0.05). Bottom: within subject variability (*n* = 9 rs1/rs2 pairs, two tailed paired *t*-test, no significance).

We assumed that a strong homotopic connectivity in healthy subjects would be largely independent of its anatomical distance (Salvador et al., 2005; see Supplementary Results) and did not observe any differences in data quality between 7 and 3 T (Supplementary Figure 5). The specificity (Grandjean et al., 2020) was determined by the ratio of the FC strength between central regions of task negative DMN [anterior cingulate (Cga) and middle cingulate cortex] and between the Cga and the middle frontal gyrus (MFG; a core region of the task positive ECN). With 7 T, we found significantly higher specificity compared to 3 T (Figure 5A, paired *t*-test, *p* < 0.05).

### 3.5. 7 T resting-state graph topology showed stronger connections within subcortical brain regions

In general, as determined by FDR, 7 T rs1 data revealed more significantly correlating connections and higher average connectivity strength relative to 3 T (Figure 5B, paired *t*-test, *p* < 0.05). For topological comparisons, the 7% strongest correlations of the average correlation matrices were represented as network graphs consisting of nodes (marking brain regions) and edges (marking functional connections between them). Those graphs can be fractionated into non-overlapping distinct communities (Supplementary Figure 6). These communities mostly resemble the ICA RSNs at a more detailed level, thereby demonstrating the high correspondence of graphs and ICA components (Supplementary Figure 7).

Next, we compared edge-specific FC strength using paired controlled network based-statistics against an independent control group (Kreitz et al., 2018; 11 healthy subjects that underwent two 3 T resting-state scans on different days, with no significant differences observed between scans at the α-level of *p* < 0.014). Thus, statistical differences beyond this value of *p* are most likely attributed to field strength differences and not by the variations of repeated measures *per se*. Based on the family wise error (*p*_FWE_) of the whole component of interconnected nodes, we assessed significant differences beyond this α-level by permutation of pairs between control and study group. To adjust for the general higher correlation values at 7 T, we normalized the data to the same mean in order to capture specific FC strength enhancements related to rather qualitative effects. As shown in Figure 5C, we demonstrate that the enhanced connectivity at 7 T was maintained for subcortical and inferior regions, particularly for the thalamus, basal ganglia and the temporal cortex (α = 0.014, *p*_FWE_ < 0.0001).

### 3.6. At 7 T, the second resting-state measurements were more harmonized between subjects

Next, we demonstrated the reliability of the resting-state measurements by cross-correlating single subject’s matrices for rs1 and rs2 without the motor task in between (*n* = 9), which were similar for each subject (Figure 5D). We detected significantly higher variability between subjects at rs1 for 7 T compared to 3 T (one factor repeated measure ANOVA, *F* = 31.63, *p* < 0.05), but no significant field strength-dependent differences at rs2, likely reflecting a higher specificity required to characterize individual resting-state networks. These networks are shaped by individual personality and experience. Their variability is based on individual connections and is therefore predestined to be visible in network graphs, but not in the more general ICA components. Additionally, 7 T rs2 measurements were more similar between subjects than the corresponding rs1 measurements (Figure 5E, top, ANOVA follow up Tukey HSD, corrected *p* < 0.05,) indicating that the shared environmental conditions (the rest inside the scanner), led to more harmonized resting-states in 7 T rs2. This effect was not observed with 3 T. The intra-subject reproducibility (*n* = 9) was not significantly different between field strengths (Figure 5E, bottom, paired *t*-test, *p* < 0.05).

### 3.7. 7 T-fMRI revealed significant modulations in the resting-state after an executed motor task

**Figure 6:**
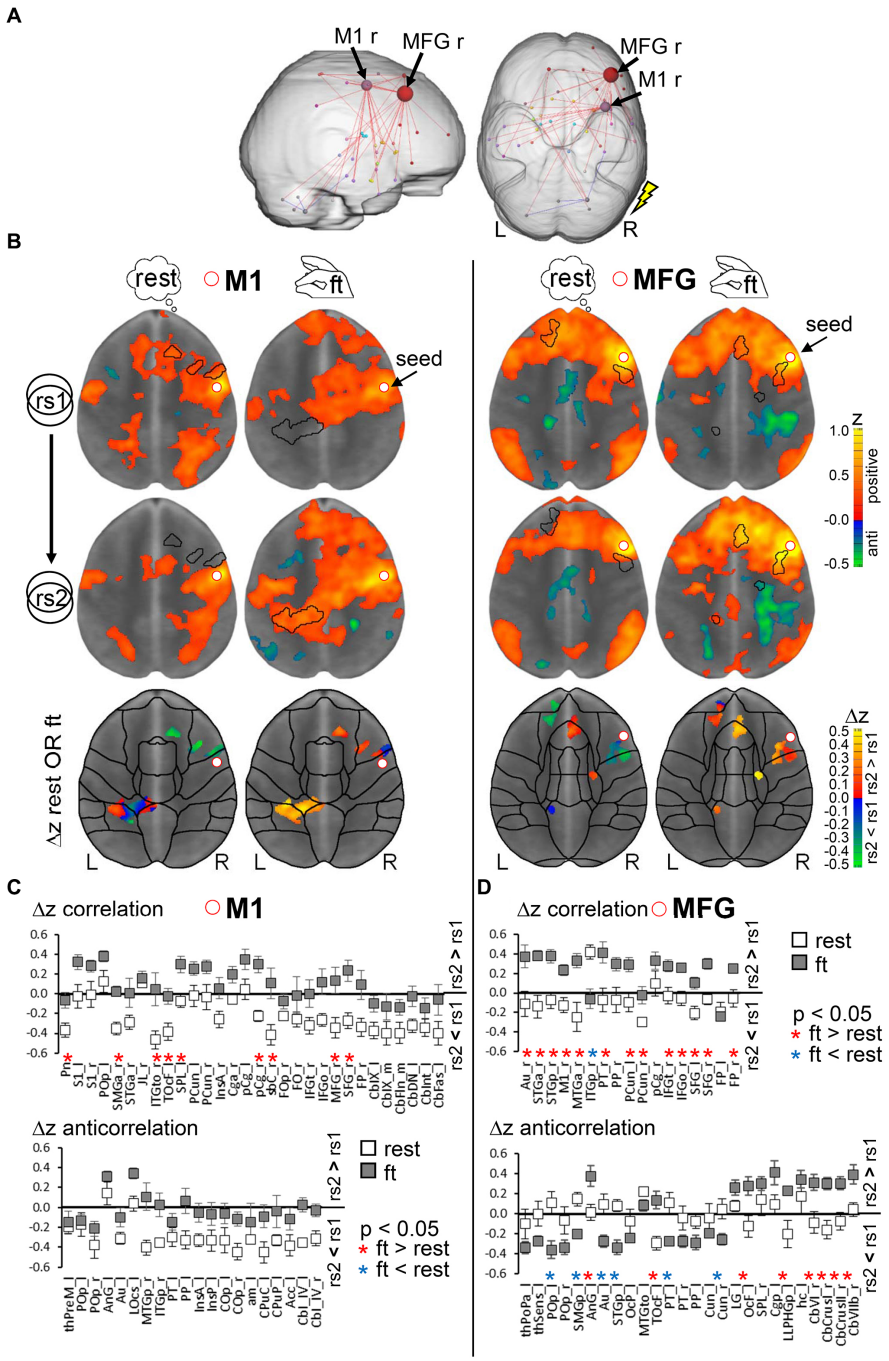
Analysis rational to detect modulations due to a prior motor task performance. **(A)** Significant graph components of enhanced resting-state connectivity strength in the second resting-state scan rs2 after finger-tapping compared to rs2 after rest (*n* = 9 per group, NBS, α = 0.01, *p*_FWE_ = 0.003). The flash indicates the stimulation side. **(B)** Combined seed region correlation (increasing red to yellow) and anticorrelation (increasing blue to green) maps using seeds in right motor cortex (M1, left) and right middle frontal gyrus (MFG, right) superimposed on MNI standard space template image. Centroid MNI coordinates of connected areas per region in rs2 of the ft-group are given Supplementary Table 2. Maps were thresholded for significance using BY-FDR (*q* = 0.05, *n* = 298 time points). The presented horizontal slice was chosen with respect to intersect the seed region (MNI *z* = 26 for M1 and *z* = 28 for MFG). Bottom: Areas with significant correlation and anticorrelation differences between rs2 and rs1 in combined for both rest- and ft. group. Displayed are average group differences per voxel (two tailed paired voxel wise *t*-test with permutation correction, *p* < 0.05). **(C,D)** Regional average differences Δ*z* (rs2 − rs1) separately for ft- and rest-group (*n* = 9 per group), but based on the same set of voxels derived from **(B)** for seed M1 **(C)** and seed MFG **(D)**. Only regions with significant voxels exceeding 1% of total region voxels were taken into account. Region-specific significance was determined by two tailed unpaired *t*-test with Benjamini-Hochberg FDR correction (^*^p < 0.05, red: ft. > rest, blue: ft. < rest). Exact *p* values and abbreviation of region names are given in Supplementary Table 3. L: left hemisphere, R: right hemisphere.

Given our findings suggesting that 7 T-fMRI detects functional signals in cortical regions with a higher tSNR-independent specificity, we next assessed short-term resting-state modulations immediately following a finger-tapping motor task. Subjects who performed the motor task between the two resting-state measurements, each at 7 and 3 T, (ft-group, *n* = 9) were compared to those remaining at rest during the whole session (rest-group, *n* = 9). Given the harmonization of the inter-subject variability we detected in rs2, we hypothesized that motor task effects might only be visible in the rs2 group comparison. Indeed, we only detected a significant component of reinforced connections in the 7 T rs2 group following the motor task [α = 0.01, pFWE = 0.003, network-based statistic (NBS) analysis, see the section 2]. Interestingly, the increased connectivity strength occurred primarily in regions relevant to the previous motor performance, namely M1 and MFG, but now ipsilateral to the stimulation side (Figure 6A). Both regions were activated bilaterally, though the M1 contralateral region was, as expected, more strongly affected (Supplementary Table 1). To investigate relationships between contralateral activation and ipsilateral connectivity modulation, we calculated the interhemispheric functional connectivity between bilateral M1 and MFG during motor performance. We found that the average time courses of both bilateral regions were significantly correlated (critical *z*-value = 0.072, *p* < 0.05, df = 744), indicating a strong interhemispheric communication. However, this correlation was significantly weaker for 3 T compared to 7 T (Supplementary Figure 8, paired *t*-test, *p* < 0.05,).

Next, we used the M1 and MFG regions at 7 T as seeds for further analysis within subjects. For both the right M1 and right MFG, we compared the seed correlation maps of rs1 and rs2 using a voxel wise paired *t*-test, and identified significant voxels (*p* < 0.05, corrected) separately for the ft- and the rest-group. Subsequently, the significant voxels of both groups were combined and assigned to atlas-based brain regions. Thus, group-specific differences in connectivity strength were assessed within the same set of voxels (Figure 6B, see Supplementary Table 3 for centroid MNI coordinates per region). Although rs1 was recorded for both groups before any intervention, the average seed correlation maps of the M1 seed showed some differences. This variation in the extension of correlating voxel around the M1 seed region might be due to individual differences in task performance and motor skills. However, the basic patterns of the MFG-seed correlation maps were comparable indicating a more stable and less individual MFG connectivity.

Finally, using this set of voxels, the average FC differences between subsequent resting-state measurements (Δz rs2–rs1) per brain region in the ft-group were controlled by an unpaired *t*-test against those in the rest-group (*p* < 0.05, corrected). Higher Δ*z* values in one group indicate a more increased (positive Δ*z*) or less decreased (negative Δ*z*) FC strength in rs2 compared to the other group (Figures 6C,D; Supplementary Table 3).

**Figure 7:**
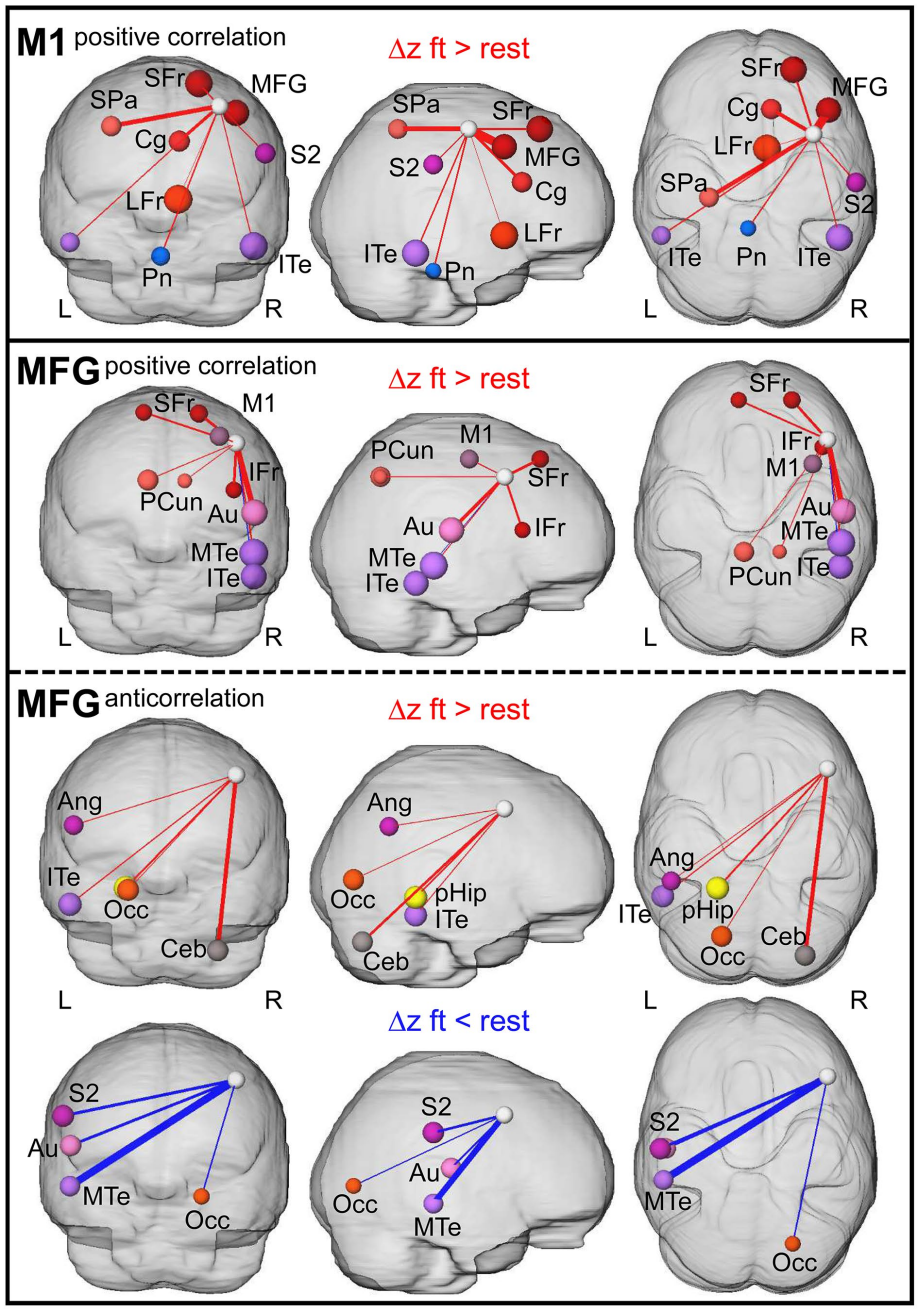
Summary of resting-state connections modulated due to prior finger-tapping. 3D visualization of graphs with nodes and edges. Node size represent absolute difference between Δ*z* (rs2−rs1) of the ft-group and the rest-group, edge thickness indicate the size of the affected area (% voxel of total region voxel). The white node represents the seed (M1 and MFG, respectively). L: left hemisphere, R: right hemisphere. Ang, angular gyrus; Au, auditory cortex; Ceb, cerebellum superior posterior lobe; Cg, cingulate cortex; IFr, inferior frontal cortex; ITe, inferior temporal cortex; LFr, limbic cortex; M1, primary motor cortex; MFG, middle frontal gyrus; MTe, middle temporal cortex; Occ, occipital cortex; Pcun, precuneus; pHip, parahippocampal gyrus; Pn, pons; S2, secondary somatosensory cortex; SFr, superior frontal cortex; and SPa, superior parietal cortex.

Ultimately, we found that, controlled against the rest-period, execution of the motor task led to significantly increased Δ*z* of M1 to ipsilateral frontal, secondary somatosensory (S2) areas, contralateral superior parietal cortex, bilateral inferior temporal cortex (ITe), and pons (Figure 6C, top; Figure 7). We did not observe any significant differences in anticorrelating Δz (rs2 − rs1 of negative correlations in the above-described set of voxels, Figure 6C, bottom). In MFG, we found increased Δz to the ipsilateral inferior frontal, auditory, medial temporal (MTe), bilateral superior frontal, and precuneus cortex (Figure 6D, top; Figure 7).

In contrast to the dominantly enhanced positive correlations with ipsilateral regions, anticorrelating Δz involved mainly contralateral regions. Here, the contralateral angular gyrus, ITe, occipital cortex (Occ), parahippocampus, and the ipsilateral superior posterior lobe of the cerebellum showed enhanced anticorrelated Δz, and contralateral S2, auditory cortex, MTe and ipsilateral Occ showed reduced anticorrelated Δz in the ft-group compared to the rest-group (Figure 6D, bottom; Figure 7, bottom). Additionally, both seeds reinforced their connectivity to each other (Figures 6C,D, 7).

Taken together, we demonstrate that a unilateral finger-tapping motor task results in contralateral activation of M1 and MFG detected by both 3 and 7 T-fMRI. However, modulation of ipsilateral M1 and MFG functional connectivity during the subsequent rest period was only detected by 7 T-fMRI.

## 4. Discussion

Here, we examined the influence of high magnetic field strength on fMRI using the BOLD signal as an indirect measure of neuronal processes in the brain. Specifically, our analysis demonstrates that, depending on the field strength, the SNR adapts through a targeted variation of the voxel size. Nevertheless, we successfully demonstrated a higher functional specificity of the BOLD signal with 7 T-fMRI.

### 4.1. Higher field strength provoke closer association of the BOLD signal with neuronal activation resulting in higher functional specificity

Under the constraint of an adjusted SNR, motor task-stimulated BOLD activation showed a nearly identical activation pattern for 3 and 7 T. Previous studies exploit the increased SNR of higher field strength and report a 300–400% increased activated volume indicating largely extended activation patterns (i.e., involved brain regions) and higher spatial sensitivity at 7 T (Yacoub et al., 2001; Schafer et al., 2008; van der Zwaag et al., 2009; Beisteiner et al., 2011). Thus, we conclude that the field strength-dependent higher SNR in earlier work is the main contributor of the higher spatial sensitivity of the BOLD response. However, even with comparable SNR and tSNR, the temporal BOLD responses showed a starkly increased signal amplitude and tCNR, which is a direct consequence of the field strength-dependent boost of the BOLD signal itself.

Functional connectivity in the resting-state can be analyzed by focusing on the 3D domain of interacting network patterns as obtained by ICA, or over time using graph-theoretical approaches. Here, we found that spatial patterns in resting-state networks derived from ICA analysis were highly consistent between 3 and 7 T. Interestingly, especially the DMN and the pVN did not only display the highest spatial similarity but also nearly identical volumes and *z* scores, supporting their general relevance in characterizing functional connectivity during rest. The task-associated cognitive and sensorimotor networks were more variable, and expressed smaller volumes but higher *z* scores at 7 T, reflecting individual characteristics of cognitive and sensorimotor resting-state networks (Bijsterbosch et al., 2017).

In contrast, graph-theoretical approaches such as MSRA, which rely on pairwise correlations of time courses, are capable of resolving functional connectivity over time. Even with lower tSNR at 7 T, we successfully identified significantly stronger functional connectivity, indicating enhanced functional specificity. This improvement was largely independent of the spatial activity distribution and characterized by tSNR-independent increased BOLD response amplitude, tCNR, and functional connectivity strength. These effects might be caused by the field strength-dependent enhanced sensitivity to the microvasculature. Several authors describe a supralinear increase in the change in relaxation rate R2* with increasing field strength for various tissues (Gati et al., 1997; Yacoub et al., 2001; Peters et al., 2007; van der Zwaag et al., 2009). This was interpreted as an increased contribution of microvascular vessels within the tissue leading to an increased specificity at 7 T (Yacoub et al., 2001, van der Zwaag et al., 2009). Since the hemodynamic response to a neural event begins in the tissue microvasculature and only then propagates to the larger draining vessels (de Zwart et al., 2005), not only the spatial but also the temporal specificity of the BOLD signal to the underlying neuronal activity is enhanced. This idea is supported by the strong correlation between local field potential (LFP) and BOLD signal changes. Notably, LFP also reflect energetically expensive synaptic activity (Logothetis et al., 2001). Furthermore, Siero et al. (2014) demonstrated that the 7 T gradient-echo BOLD signal is strongly correlated with the underlying electrophysiology, and thus it corresponds well with the neuronal activity. Therefore, we conclude that the increased functional specificity at 7 T was caused by the closer temporal and spatial association of the BOLD signal with the underlying neuronal activation in the brain. However, due to the larger voxel size, the increased tSNR at 3 T is partially negated by an increased partial volume effect, i.e., the detection of a mixed signal of different tissues within one voxel volume. To counteract this effect, the voxel size of 2 mm^3^ was chosen small enough to minimize partial voluming and ensure optimal statistical specificity and sensitivity (Weibull et al., 2008). Additionally, the resting-state time course correlations were restricted to grey matter in order to reduce the occurrence of tissue mixing in voxel volumes. Nevertheless, we cannot exclude an impact of the reduced partial volume effect on the higher specificity of 7 T fMRI.

### 4.2. Higher functional specificity enables the detection of a functional correlate to the offline replay of neuronal motor activity

To exploit the increased functional specificity of 7 T-fMRI, we investigated resting-state modulations after a motor movement paradigm. Even under the constraint of a completely data-driven analysis, we identified the cortical regions M1 and MFG, activated by the motor performance, as the core regions that were specifically modulated in the subsequent resting-state. Importantly, these modulations could not be detected with 3 T, providing proof of concept, that 7 T detects resting state-specific modulations, directly driven by neuronal activity that is reflected as specific functional connectivity. We propose that these modulations represent a functional correlate to the offline replay of neuronal firing sequences, detected by using invasive electrophysiology, e.g., for the mouse auditory cortex after an auditory task (Yu et al., 2021) or the human motor cortex after a motor task (Eichenlaub et al., 2020).

### 4.3. Replayed neuronal firing sequences immediately initiate the modulation of functional memory circuits

In this study, we present a neuronal replay visible in brain-wide functional connectivity modulations, complementing the findings of replay of electrophysiological neuronal firing sequences (Eichenlaub et al., 2020) and probabilistic fMRI activation patterns (Wittkuhn and Schuck, 2021) in humans. In contrast to those previous studies, we were able to characterize function and nature of the replay. The majority of modulated functional connectivity did not resemble that of brain regions participating in previous finger tapping-associated motor processing, but instead corresponded to frontal–parietal and temporal circuits involved in memory processing and consolidation (Wager and Smith, 2003; Harrison and Tong, 2009; Vilgis et al., 2014; Panichello and Buschman, 2021). This was unexpected, since finger-tapping is presumed to predominantly lead to motor area activation not overlapping with working memory regions (Wesley and Bickel, 2014). Moreover, task-induced neuronal representations of brain activity were found to be inherent in the resting-state (Mennes et al., 2010), reflecting at least some task-induced brain activity patterns during rest (Kusano et al., 2015; Niu et al., 2020). However, the offline replay correlate we identified by 7 T-fMRI, indicates that replayed neuronal firing sequences immediately initiate the modulation of functional connectivity related to memory circuits. This refers dominantly to working memory such as the frontal-temporal parietal circuit but also to regions with central roles in autobiographic (here the precuneus; Mazzoni et al., 2019) and episodic (here the medial temporal gyrus; Rugg and Vilberg, 2013) memory.

### 4.4. The specific modulation of anticorrelated MFG connections support the involvement of early memory consolidation

We also detected modulated anticorrelations in MFG, an important region in memory processing, but not in M1 that dominantly reflects the former motor activation. The switch between task-positive and task-negative (i.e., anticorrelated) resting-state networks is an important feature in memory consolidation (Keller et al., 2015; Piccoli et al., 2015; Meskaldji et al., 2016; Franzmeier et al., 2017). The MFG is the core region of the task positive executive control network which is also implicated in working memory processing (Olesen et al., 2004; Osaka et al., 2004), thereby acting as the main antagonist to the task negative DMN (McKiernan et al., 2003). We found both, enhanced and reduced anticorrelated functional connectivity strength. This is in line with Piccoli et al. (2015), who suggest a dynamic switch of FC between task positive and task negative (i.e., the DMN) brain networks during memory consolidation. In general, enhanced anticorrelation is associated with better memory performance (Keller et al., 2015; Meskaldji et al., 2016; Franzmeier et al., 2017). In this context we found enhanced anticorrelation to MFG in contralateral regions specifically important for memory consolidation such as the angular gyrus (Burianova et al., 2012; Yuksel et al., 2018), the inferior temporal gyrus (Tomita et al., 1999; Axmacher et al., 2008), the occipital fusiform gyrus (Kundu et al., 2015; Tambini and D’Esposito, 2020), and the parahippocampal gyrus (Dickerson and Eichenbaum, 2010; Ward et al., 2014). Additionally, the ipsilateral superior posterior cerebellum, namely lobule VI, Crus II, Crus I, and lobule VIIb, were anticorrelated to the MFG. The cerebellum is not only involved in motor control but also in cognitive and emotional functions including working memory (Middleton and Strick, 1994; Baillieux et al., 2008; Keren-Happuch et al., 2014; Guell et al., 2018). Recent human studies revealed a functional topography of the cerebellum with distinct representation of different sensorimotor and cognitive functions (Guell et al., 2018; Ashida et al., 2019). Some of these functional compartments overlap, such as language and emotion (Guell et al., 2018), motor and sensory, as well as language and working memory (Ashida et al., 2019), indicating an integrative function of these overlapping areas. However, none of these studies investigated the overlap of motor response and working memory, although the description of the activated areas for both tasks separately indicate the possibility of such an overlap. Consistently, finger movement activated the ipsilateral anterior lobule V and VI, while activation of areas involved in working memory were located in the right lobule VI and, depending on the memory load, extended to right CrusII and VIIb (Desmond et al., 1997; Guell et al., 2018; Ashida et al., 2019). In the present work, we found enhanced anticorrelation to the memory related MFG for exactly those cerebellar regions (right lobule VI, CrusII, and VIIb) that count for working memory. However, additionally to the described motor related lobule V and VI these regions were also activated during task performance. Presuming a spatial overlap of cerebellar motor control and memory function support the hypothesis that the replay of task related activity during rest is directly coupled to working memory circuits highlighting the integrative role of the RS itself in continuous integration of behavior and memory.

Reduced anticorrelations are more difficult to interpret. However, it stands out, that those structures that show reduced anticorrelation on the left hemisphere were strengthened in their positive correlation on the ipsilateral side (Au, S2, and MTE) and vice versa (ITE). Maybe this interhemispheric interplay promotes the lateralized region’s specific function in memory consolidation. This functional memory related lateralization has been frequently observed with memory task induced activation patterns (Wager and Smith, 2003) and recently also for anticorrelated connectivity strength (Meskaldji et al., 2016).

### 4.5. The switch between hemispheres of M1 functional connections might reflect an encoding/retrieval asymmetry

Additionally, the switch between hemispheres regarding M1 might reflect a hemispheric encoding/retrieval asymmetry (HERA; Tulving et al., 1994; Habib et al., 2003; Andreau and Torres Batan, 2019; Papp et al., 2019). This model describes a stronger activation of the left prefrontal cortex during encoding of information into memory and the right prefrontal cortex being more active during retrieval of memory information. This could be recently verified for verbal memory tasks, but not for visual (Andreau and Torres Batan, 2019) and was also observed in rats performing a novel object recognition test (Papp et al., 2019). In the present study, a HERA like switch between hemispheres was observed for M1, which was activated on the left hemisphere during finger-tapping (respective encoding) and showed enhanced connectivity strength to memory related regions on the right hemisphere in subsequent rest, which can be presumed to be an early maintenance phase of memory consolidation.

### 4.6. Limitations

Some limitations in this study should be addressed. First, the sample size of nine participants per group is relatively low compared to recent neuroscience studies. To enhance statistical power we compared paired differences between resting state measurements (Δz). Additionally, the proposed statistical approach is highly controlled in individual (rs2 vs. control rs1), group (ft vs. control rest group), and multiple comparison (voxel wise using BY-FDR and region wise using BH-FDR) in order to reduce the occurrence of false positive results. We cannot exclude that a sufficiently increased sample size in the 3 T study would also allow detecting motor cortex offline replay modulations despite the lower neuronal specificity. Even if so, this does not contradict our finding, that the enhanced specificity of high field fMRI facilitate the identification of tiny resting-state modulations.

Second, SNR of 3 and 7 T measurements were adjusted only by adapting the voxel size ratio according to the inverse field strength ratio without detailed adaptation of the protocols. The actual field strength dependent SNR is also influenced by the RF coil and can be higher (Pohmann et al., 2016). In fact, the resulting 3 T SNR was overcompensated and higher than the 7 T SNR. Additionally, the accuracy of SNR and CNR determination was limited. Both parameters were calculated using the background as reference, which is hampered by the unequally distributed g-factor noise across the image. However, methods to more accurately determine SNR and CNR are laborious, expensive and not easily applicable to clinical routine or biological practice. Therefore, we chose the commonly used method of Magnotta et al. (2006) and defined the background regions as the largest contiguous region outside the brain tissue mask with less intensity than the 5% quantile of the brain. While this is not sufficient, it at least eliminates larger artifacts and reduces variability.

Third, we provide no experimental evidence for the relationship of modulated motor cortex (M1 and MFG) connections to memory consolidation. This should be part of future studies.

## 5. Conclusion

In conclusion, we demonstrated, for the first time, the application of 7 T-fMRI to detect a highly specific neuronal activity in the resting-state that is directly linked to the execution of a motor task. Keeping SNR constant, spatial distribution of functional activation and connectivity did not differ between field strength. However, the temporal fluctuation of the BOLD signal, mirroring the underlying neuronal activity, showed enhanced specificity with higher field strength that is not triggered by cortical tSNR. The enhanced specificity of 7 T-fMRI was sufficient to capture resting-state modulations following a simple finger-tapping motor task assumed to be directly related to the offline replay of neuronal firing sequences. Although the finger-tapping motor task should neither activate nor enhance the connectivity of memory related brain regions the observed M1 and MFG modulations dominantly concern memory and learning circuits—including well known mechanisms of memory consolidation such as anticorrelation of task negative circuits and encoding/retrieval asymmetry. We hypothesize that this short-term initiation of memory circuits accompanying the neuronal offline replay is a principle mechanism preparing but not necessarily determining later successful memory consolidation. Thus, in combination with our sophisticated analysis workflow, the higher functional specificity of 7 T fMRI might open the door to more detailed and sophisticated basic research aimed at understanding human memory consolidation but also neurological diagnostics in clinical routine in the very near future.

## Data availability statement

Unless there is no ethical approval conflict, the raw data supporting the conclusions of this article will be made available by the corresponding author upon reasonable request.

## Ethics statement

The studies involving human participants were reviewed and approved by Ethical approval (189-15B) provided by the local ethics committee of FAU. The study adhered to the tenets of the Declaration of Helsinki. The patients/participants provided their written informed consent to participate in this study.

## Author contributions

SK: conceptualization (neuroscience), software, data analysis, visualization, and writing—original draft. AM: conceptualization (physics), participant recruitment, data acquisition, and writing—review and editing. LK: participant recruitment, data analysis, and writing—review and editing. JR: data acquisition and writing—review and editing. AN and FL: conceptualization (physics) and writing—review and editing. MU and AD: supervision, conceptualization (radiology), and writing—review and editing. AH: supervision, conceptualization (neuroscience), methodology, project administration, and writing—review and editing. All authors contributed to the article and approved the submitted version.

## Funding

AD is supported by the Deutsche Forschungsgemeinschaft (DFG, German Research Foundation) project number 460333672 – CRC 1540 Exploring Brain Mechanics (subproject A02).

## Conflict of interest

SK and AH are CEOs of BioCom GbR, a company that sells MagnAn analysis software. They did not get any financial benefit from providing the software for the study.

The remaining authors declare that the research was conducted in the absence of any commercial or financial relationships that could be construed as a potential conflict of interest.

## Publisher’s note

All claims expressed in this article are solely those of the authors and do not necessarily represent those of their affiliated organizations, or those of the publisher, the editors and the reviewers. Any product that may be evaluated in this article, or claim that may be made by its manufacturer, is not guaranteed or endorsed by the publisher.

## Footnotes

^1^ https://www.fmrib.ox.ac.uk/datasets/brainmap+rsns/

^2^ http://greiciuslab.stanford.edu/resources

## Supplementary material

SUPPLEMENTARY DATA SHEET 1 A list of all abbreviations used in this article is provided in Appendix 1.
