# Supplementary material for: 3T vs. 7T fMRI: capturing early human memory consolidation after motor task utilizing the observed higher functional specificity of 7T
Source: Front Neurosci. 2023 Aug 10;17:1215400. doi: 10.3389/fnins.2023.1215400 (PMC10448826; doi:10.3389/fnins.2023.1215400)
Supplement: SUPPLEMENTARY DATA SHEET 1 — A list of all abbreviations used in this article is provided in Appendix 1. [file Data_Sheet_1.PDF]

Appendix 1: Abbreviations

Data acquisition related:

BOLD: Blood Oxygen Level Dependent

FOV: Field Of View

GE-EPI: Gradient Echo-Planar Imaging sequence

GRAPPA: GeneRalized Autocalibrating Partially Parallel Acquisitions

gre-field: gradient echo field

HRF: Hemodynamic Response Function

fMRI: functional Magnet Resonance Imaging

MP2RAGE: Magnetization Prepared 2 Rapid Gradient Echo sequence

MRI: Magnet Resonance Imaging

rs-fMRI: resting state functional Magnet Resonance Imaging

TA: Acceleration Time (GRAPPA)

TE: Echo Time

TR: Repetition Time

Analysis related:

FC: Functional Connectivity

ft: finger tapping

FWHM: full Width Half Max

MNI: Montreal Neurological Institute

ROI: Region Of Interest

rs1: resting state 1

rs2: resting state 2

Resting state networks:

aSN: anterior Saliency Network

AuN: Auditory Network

DMN: Default Mode Network

ECN: Executive Control Network

LECN: Left Executive Control Network

lVN: lateral Visual Network

oVN: occipital Visual Network

pVN: primary Visual Network

RECN: Right Executive Control Network

RSN: Resting State Network

SMN: SensoriMotor Network

Quality measures:

CNR: Contrast to Noise Ratio

EFC: Entropy Focus Criterium

FBER: Foreground to Background Energy Ratio

Gcorr: Global correlation

MDI: Median Distance Index

SNR: Signal to Noise Ratio

tCNR: temporal Contrast to Noise Ratio

tSNR: temporal Signal to Noise Ratio

zDVARS: standardized (z) temporal derivative (D) of root mean square variance (VARS)

Task response parameter:

AP: Activation Probability

AV: Activated Volume

PH: Peak Height (of response amplitude)

PS: Peak Symmetry (of response amplitude)

PT: Peak Time (of response amplitude)

PW: Peak Width (of response amplitude)

Statistics:

ANOVA: ANalysis Of VAriance

BH-FDR: False Discovery Rate according to Benjamini and Hochberg

BY-FDR: False Discovery Rate according to Benjamini and Yekutieli

df: degrees of freedom

FDR: False Discovery Rate

GLM: General Linear Model

ICA: Independent Component Analysis

NBS: Network Based Statistics

MSRA: Multi Seed Region Analysis

PCA: Principle Component Analysis

PC: Principle Component

pFWE: probability of Family Wise Error

pNBS: paired Network Based Statistics

SCM: Seed Correlation Map

SPM: Statistical Parametric Map

TFCE: Threshold Free Cluster Enhancement

Other:

LFP: Local Field Potential
