# Supplementary material for: 3T vs. 7T fMRI: capturing early human memory consolidation after motor task utilizing the observed higher functional specificity of 7T
Source: Front Neurosci. 2023 Aug 10;17:1215400. doi: 10.3389/fnins.2023.1215400 (PMC10448826; doi:10.3389/fnins.2023.1215400)
Supplement: Supplementary file 5 [file Data_Sheet_2.pdf]

Supplementary Material

Content:

Supplementary Results:

- Identification of RSNs
- Quality assessment of RS graphs

Supplementary Figures 1 – 8

Supplementary Figure 1: BOLD response parameters describing the BOLD signal response amplitude.

Supplementary Figure 2: Spatial distribution of tSNR throughout the brain for the first resting-state.

Supplementary Figure 3: Regional activation probability due to a finger-tapping motor task.

Supplementary Figure 4: Identification of resting-state networks derived from 20-component group ICA.

Supplementary Figure 5: Quality assessment of resting state matrices derived from multi-seed-region analysis.

Supplementary Figure 6: Communities of resting-state MSRA graphs.

Supplementary Figure 7: Brain regions contributing to RSNs derived from group ICA aggregate components presented as nodes superimposed on MSRA community graphs.

Supplementary Figure 8: Interhemispheric functional connectivity during task performance.

Supplementary Tables 1 – 3

(provided as separate data files)

Supplementary Table 1.xlsx: BOLD response parameters to finger tapping motor stimulation obtained with 7T and 3T fMRI

Supplementary Table 2.xlsx: Average MNI coordinates of activated and connected areas within brain regions.

Supplementary Table 3.xlsx: Brain regions containing voxels of the "Δz rest OR ft" mask of the M1 and MFG network, respectively.

Supplementary Results

Identification of RSNs

Using various similarity measures to two different sets of templates (Smith and Stanford, see Materials and Methods) we reliably could identify nine RSNs (Supplementary Figure 4A). Using the criteria of maximum similarity converging in both directions (template to ICA and ICA to template), five RSNs could be unambiguously assigned to both, the Smith Template and the Stanford template. These RSNs were the auditory (auN), primary visual (pVN), occipital visual (oVN), and left and right executive control (LECN, RECN, respectively) networks. Sensorimotor (SMN) and anterior Saliency (aSN) were identified solely according to the Stanford template. The default mode network (DMN) was assigned with high similarity to the corresponding Smith template, but the best match of the Stanford template to this ICA component by the similarity measures was the precuneus network. The precuneus network and the DMN strongly overlap and may not be separated accordingly using 20 ICA components as used for both the Smith template and our data. However, the Stanford template was created using 30 ICA components, therefore the DMN was separated into a ventral and a dorsal part and additionally the precuneus network was detected. The similarity to the templates varied greatly between RSNs, indicating RSN specific variations in spatial reliability. Highest spatial correlation to the Smith template was found for the 3T pVN (fisher's $z = 0.896$), the least similarity occurred for the 7T AuN (fisher's $z = 0.234$) with a median of fisher's $z=0.484$ or all identified RSNs (Supplementary Figure 4B). The spatial overlap of the binarized RSNs to the Stanford template, i.e. the proportion of template ROIs covered by the RSNs, was also highest for the 3T pVN (overlap ratio = 0.997) and lowest for the 7T lVN (overlap ratio = 0.181) (Supplementary Figure 4C). The lVN of the Stanford template covers dominantly regions involved in visuospatial processing, whereas the lVN of the Smith

template is located lateral occipital and associated more to the higher visual areas. The median overlap was nearly 60 % (overlap ratio = 0.595). According to the greater extension of some binarized 3T RSNs most overlap values were also higher for 3T. The similarity according to the Jaccard index, which normalizes for the RSN size, showed no difference for the majority of RSNs between 3T and 7T (Supplementary Figure 4D). The same could be observed regarding the spatial correlations to the Smith templates (Supplementary Figure 4B). Thus, variations in similarity to the template were not dependent on field strength but rather on the intrinsic variability of the RSNs themselves.

Quality assessment of RS graphs

Quality assessment was initially demonstrated by the dominant connectivity strength between bilateral regions, visible as diagonal lines in the correlation matrices with brain regions sorted by hemisphere (Supplementary Figure 5A). Mathematically, the data quality was assessed by the linear fit of the percentage correlation rank of corresponding bilateral brain regions in dependence on their anatomical distance (Supplementary Figure 5B). Though the percentage rank difference per cm anatomical distance was near 0 (-0.25% for 3T and -1.3 % for 7T), the distance dependency was significantly higher for 7T data (Supplementary Figure 5b,c, paired t-test, $p<0.05$). However, no field strength specific significant differences in the average correlation rank of all corresponding bilateral brain regions could be observed indicating comparable basic data quality for both field strength (Supplementary Figure 5D).

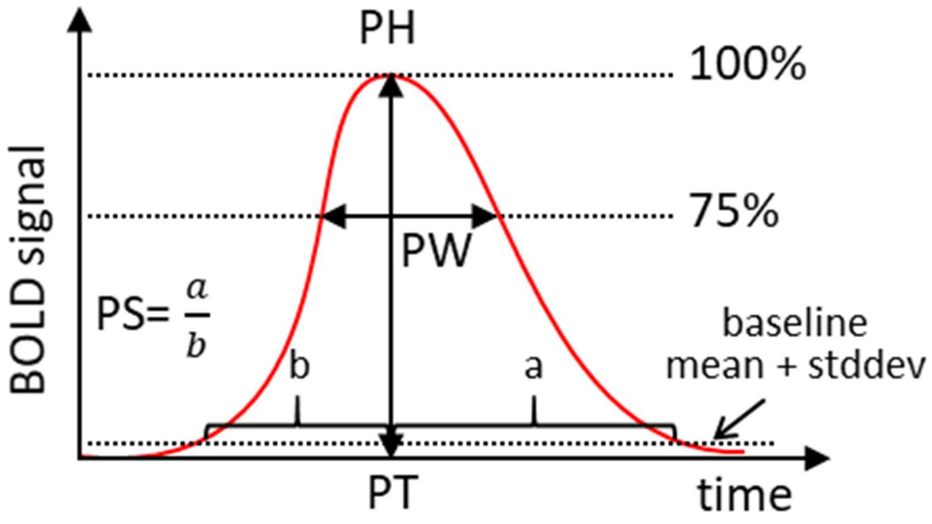

Supplementary Figure 1: BOLD response parameters describing the BOLD signal response amplitude.

PH: Amplitude (peak) height: maximum % BOLD signal change. PT: Time to peak: time point of maximum % BOLD signal change, expressed in sec after stimulation onset. PW: Amplitude width: all time points above 75 % of maximum % BOLD signal change, expressed in sec. PS: Amplitude symmetry: ratio of time points after and before time to peak above whole baseline (before and after stimulation period) mean + standard deviation. Values greater than 1 indicate tailing of the response amplitude, and values lower than 1 mean a slower increase to the maximum.

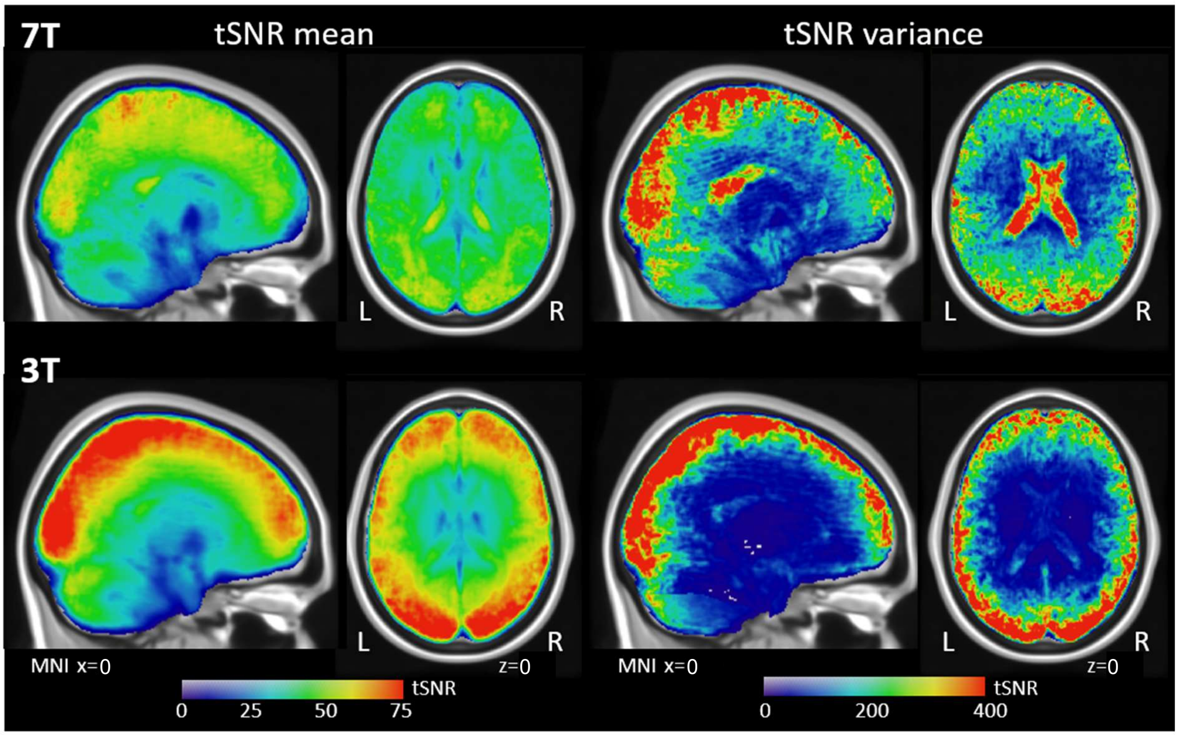

Supplementary Figure 2: Spatial distribution of tSNR throughout the brain for the first resting-state.

Top: 7T rs1 scan, bottom: 3T rs1 scan. Average (left) and variance (right) heat maps are superimposed on MNI standard space template. L: left hemisphere, R: right hemisphere.

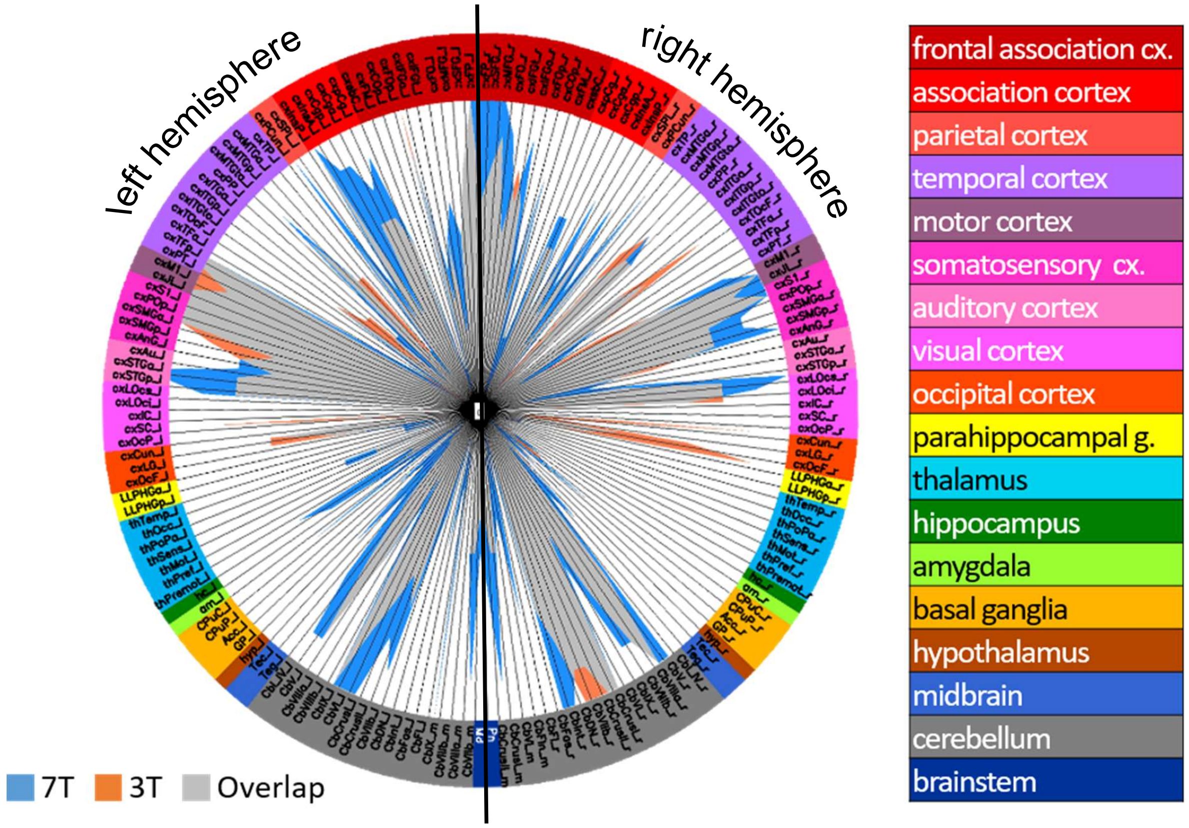

Supplementary Figure 3: Regional activation probability due to a finger-tapping motor task.

Each participant was scanned with 7T and 3T, respectively. The activation probability is the proportion of participants that show motor task induced activation in a respective brain region.

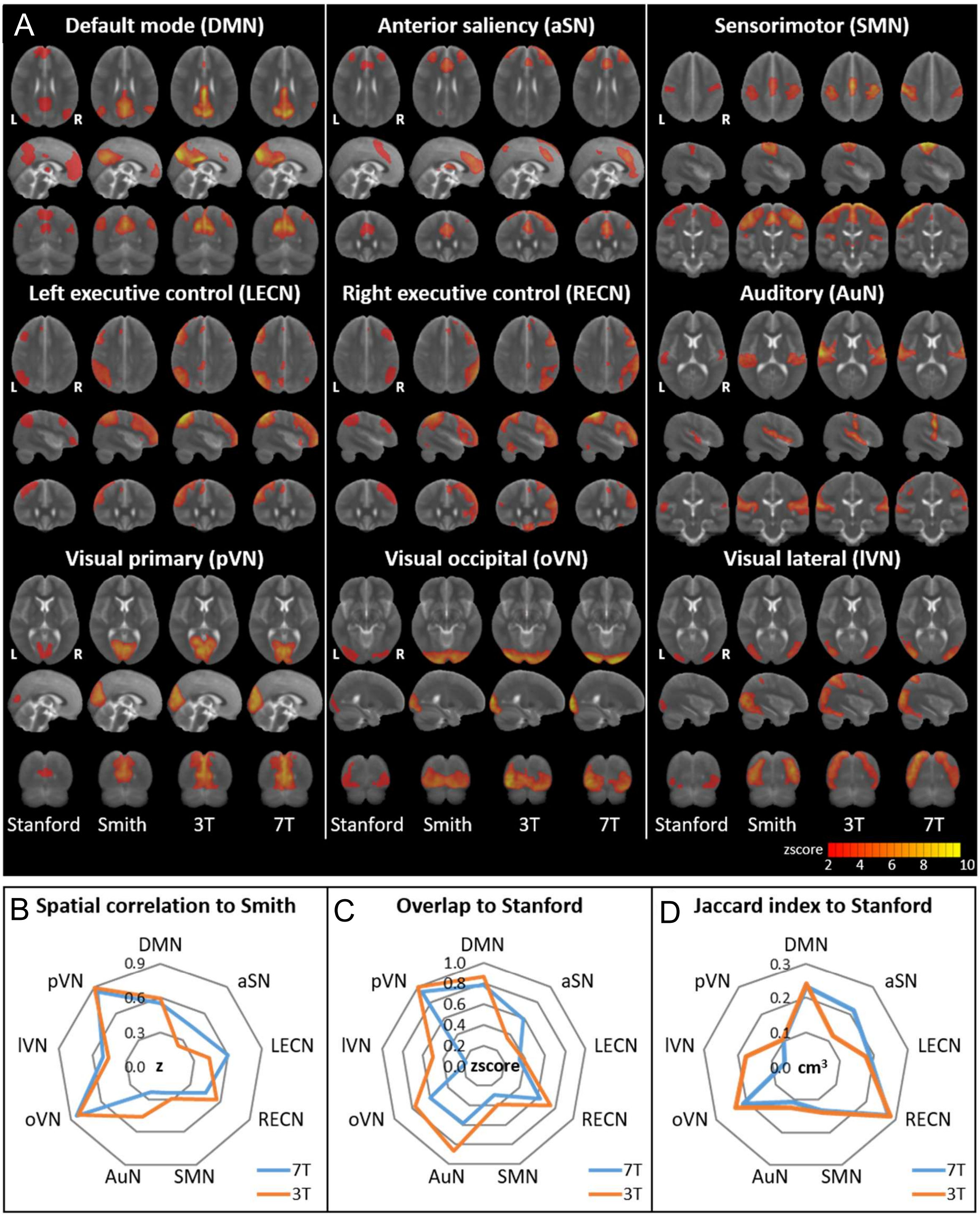

Supplementary Figure 4: Identification of resting-state networks derived from 20-component group ICA.

(A) Public available resting-state network (RSN) templates (first column of each set: Stanford, second column: Smith, for details see Methods) and group ICA aggregate components (zscore maps) of 3T (third column) and 7T (fourth column) resting-state rs1 scans. 3T and 7T zscore maps were thresholded at zscore=2 (corresponding to $p<0.05$, uncorrected). The Smith template was thresholded accordingly to match the 3T zscore map best. The Stanford template is provided binarized. The 3 most informative orthogonal slices for each set and RSN was visualized superimposed on the MNI standard space template image. (B) Identification of RSNs via spatial cross correlation of 3T and 7T ICA zscore maps to the Smith template. (C) Identification of RSNs via spatial overlap of thresholded (zscore > 2) and binarized 3T and 7T ICA zscore maps with Stanford template. (D) Similarity of thresholded (zscore > 2) and binarized 3T and 7T ICA zscore maps with Stanford template assessed via the Jaccard index.

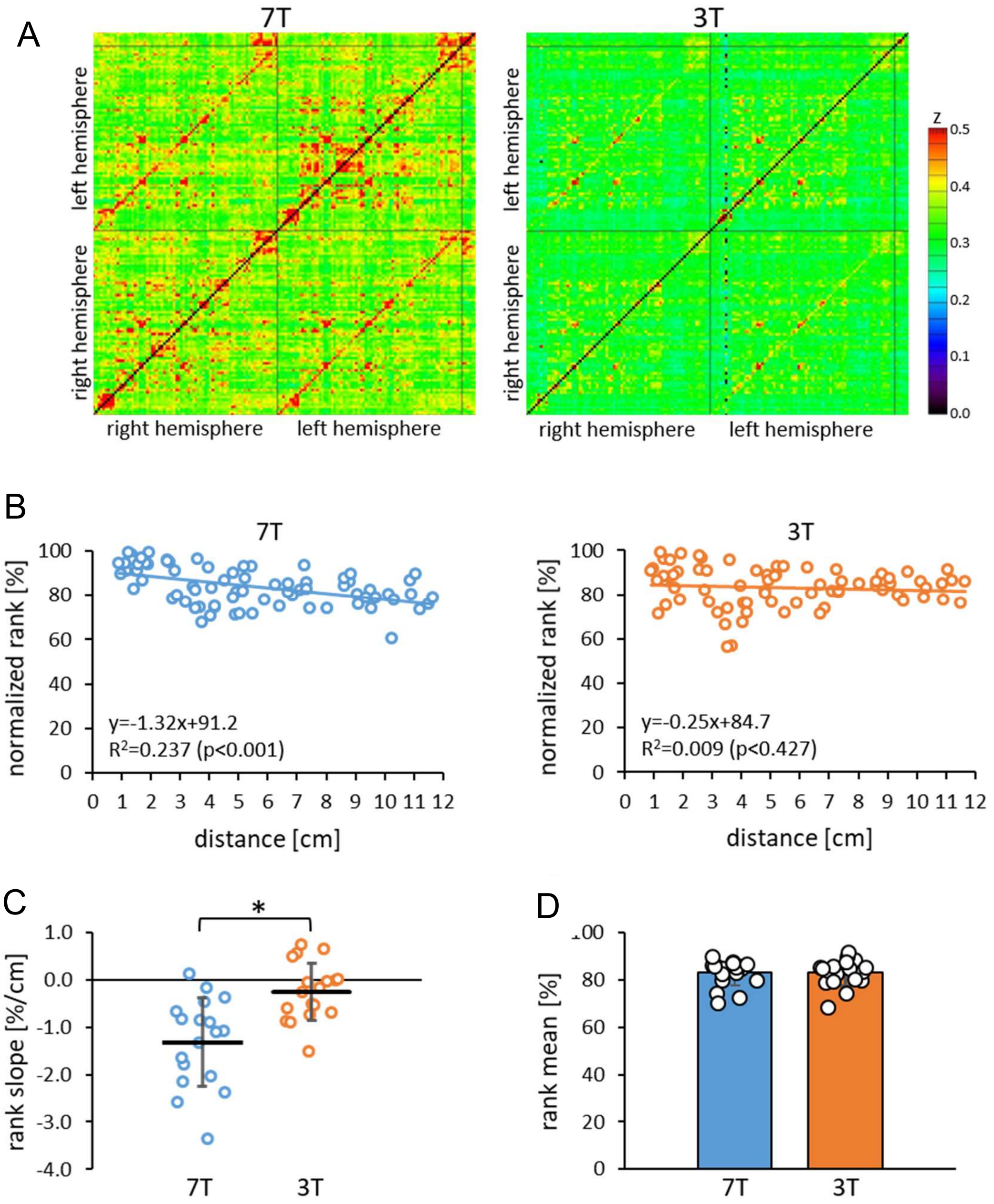

Supplementary Figure 5: Quality assessment of resting state matrices derived from multi-seed-region analysis.

(A) Average MSRA correlation matrices resulting from rs1 scans using 7T (left) and 3T (right) fMRI. (B) Ranked average correlation of bilateral brain regions across hemispheres dependent on their anatomical distance per subject rs1 scan using 7T (left) and 3T (right) fMRI, including linear trend lines. (C) Slope of the linear trend line described in (B) per subject (n=18, *$p < 0.05$, paired t-test). (D) Average correlation rank of all bilateral regions per subject. No significant differences between 7T and 3T were observed (n=18, paired t-test).

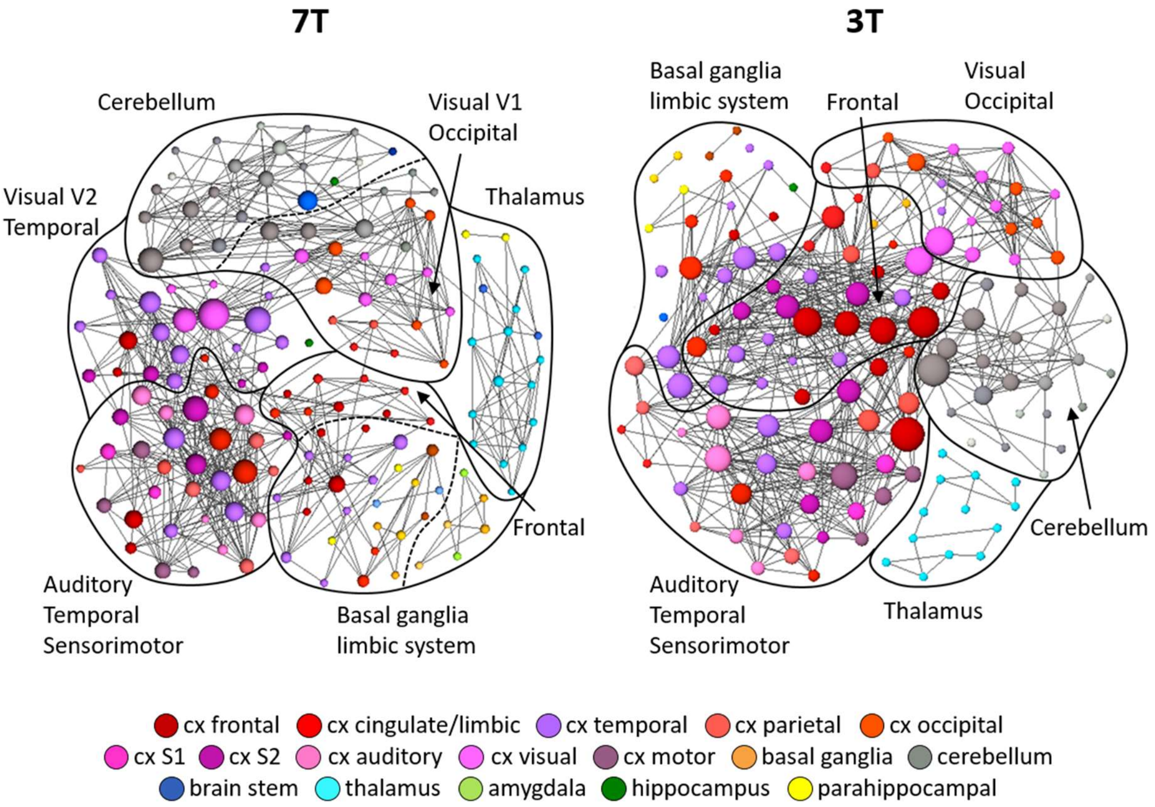

Supplementary Figure 6: Communities of resting-state MSRA graphs.

Average 7T (left) and 3T (right) first resting-state rs1 scans, normalized to a density of 7% of all possible connections are shown. Communities were detected using the Blondel algorithm and marked by surrounding solid (community level 1) and dashed (community level 0) lines. Node size represent their degree (i.e. the number of connections per node). Graphs were visualized using a forced based algorithm.

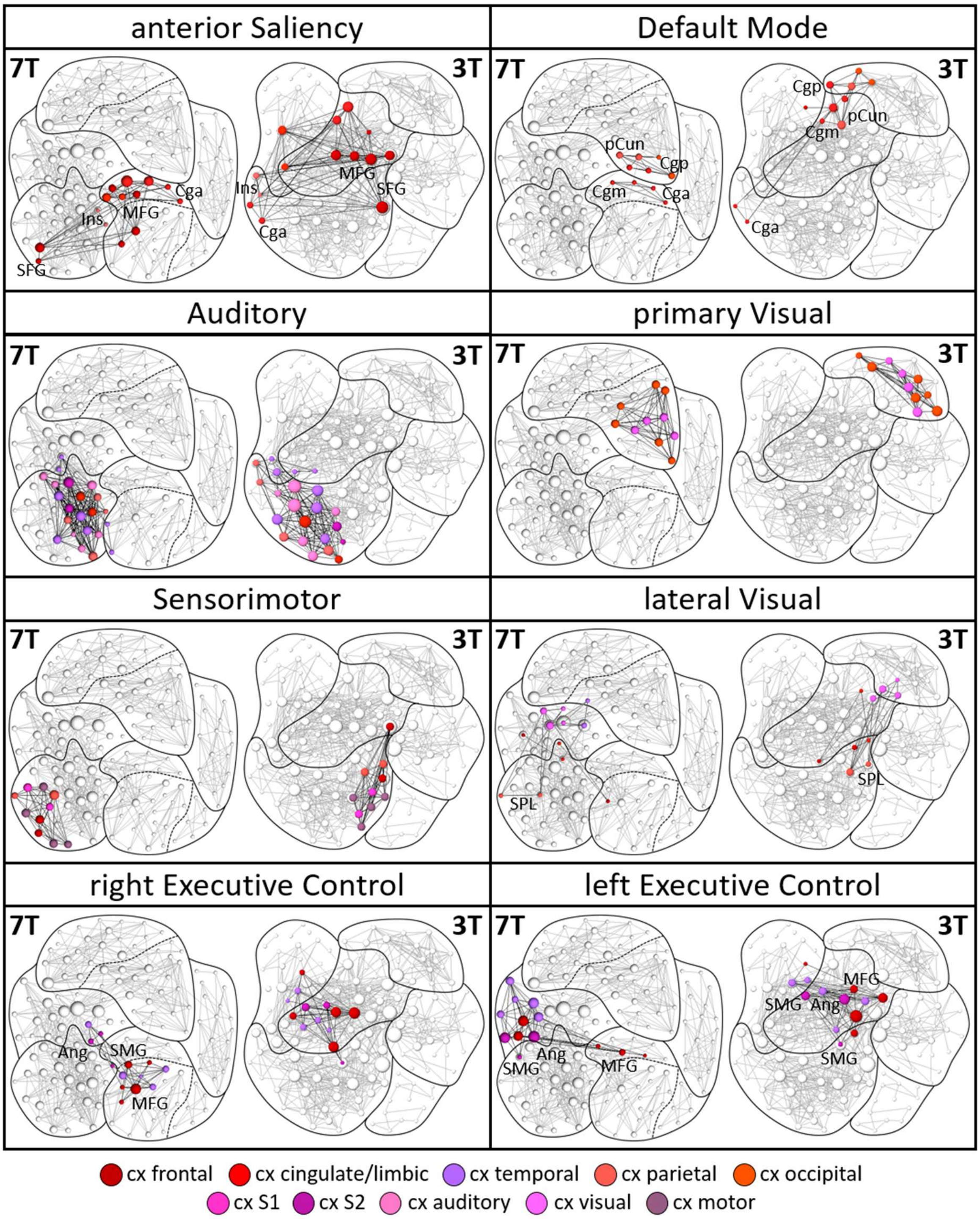

Supplementary Figure 7: Brain regions contributing to RSNs derived from group ICA aggregate components presented as nodes superimposed on MSRA community graphs.

Node size code for their degree in a complete graph normalized to 7% density. The occipital visual RSN largely overlaps with the primary visual RSN. The dominant region characterizing solely the occipital visual RSN is the occipital pole, located in the same community as and closely related to the primary visual RSN. Therefore, as it hardly represents a graph theoretical subnetwork, the occipital RSN is not shown. Ang: angular gyrus, Cga: anterior cingulate cortex, Cgm: middle cingulate cortex. Cgp: posterior cingulate cortex, Ins: insula, MFG: middle frontal gyrus, pCun: precuneus, SMG: supramarginal gyrus, SFG: superior frontal gyrus.

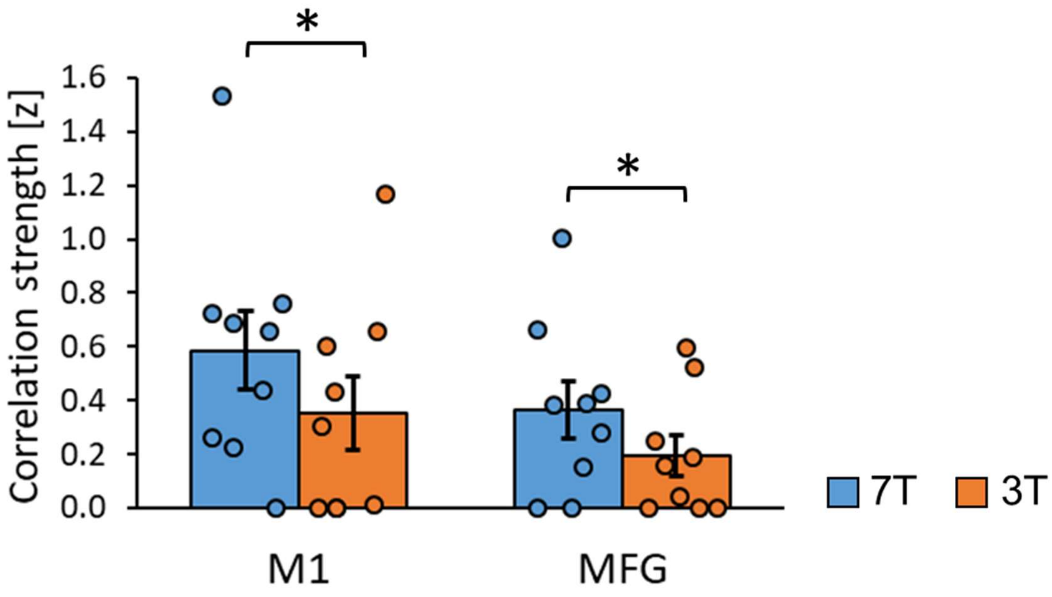

Supplementary Figure 8: Interhemispheric functional connectivity during task performance.

Functional connectivity was assessed via correlation of average regional time courses after regressing out the global mean time course of all activated regions to eliminate the stimulation driven response. * paired t-test, $p<0.05$, $n=9$
